# Ex vivo processing for maturation of *Arabidopsis* KDEL-tailed cysteine endopeptidase 2 (AtCEP2) pro-enzyme and its storage in endoplasmic reticulum derived organelles

**DOI:** 10.1007/s11103-013-0157-6

**Published:** 2013-11-28

**Authors:** Georg Hierl, Timo Höwing, Erika Isono, Friedrich Lottspeich, Christine Gietl

**Affiliations:** 1 Center of Life and Food Sciences Weihenstephan, Lehrstuhl fuer Botanik, Technische Universitaet Muenchen, Emil-Ramann-Str. 4, 85350 Freising, Germany; 2 Center of Life and Food Sciences Weihenstephan, Department of Plant Systems Biology, Technische Universitaet Muenchen, Emil-Ramann-Str. 4, 85350 Freising, Germany; 3 Max Planck Institute of Biochemistry, Protein Analysis, 82152 Martinsried, Germany

**Keywords:** Ricinosomes, ER-bodies, Programmed cell death, Developmental tissue remodeling, Cell wall degradation

## Abstract

Ricinosomes are specialized ER-derived organelles that store the inactive pro-forms of KDEL-tailed cysteine endopeptidases (KDEL-CysEP) associated with programmed cell death (PCD). The *Arabidopsis* genome encodes three KDEL-CysEP (AtCEP1, AtCEP2, and AtCEP3) that are differentially expressed in vegetative and generative tissues undergoing PCD. These *Arabidopsis* proteases have not been characterized at a biochemical level, nor have they been localized intracellularly. In this study, we characterized AtCEP2. A 3xHA-mCherry-AtCEP2 gene fusion including pro-peptide and KDEL targeting sequences expressed under control of the endogenous promoter enabled us to isolate AtCEP2 “ex vivo”. The purified protein was shown to be activated in a pH-dependent manner. After activation, however, protease activity was pH-independent. Analysis of substrate specificity showed that AtCEP2 accepts proline near the cleavage site, which is a rare feature specific for KDEL-CysEPs. mCherry-AtCEP2 was detected in the epidermal layers of leaves, hypocotyls and roots; in the root, it was predominantly found in the elongation zone and root cap. Co-localization with an ER membrane marker showed that mCherry-AtCEP2 was stored in two different types of ER-derived organelles: 10 μm long spindle shaped organelles as well as round vesicles with a diameter of approximately 1 μm. The long organelles appear to be ER bodies, which are found specifically in Brassicacae. The round vesicles strongly resemble the ricinosomes first described in castor bean. This study provides a first evidence for the existence of ricinosomes in *Arabidopsis*, and may open up new avenues of research in the field of PCD and developmental tissue remodeling.

**Electronic supplementary material:**

The online version of this article (doi:10.1007/s11103-013-0157-6) contains supplementary material, which is available to authorized users.

## Introduction

Programmed cell death (PCD) is a genetically determined, highly regulated process in all multicellular organisms. PCD is a prerequisite for the successful development of plants. It is involved in the development of generative tissues during micro- and macro-gametogenesis, in seed and fruit maturation, and in seed detachment and dispersal. It occurs in the development of vegetative tissues such as root cap, aerenchyma or wood formation (Beers 1997; Hadfield and Bennett 1997). Furthermore, PCD is an integral part of the responses to abiotic stress and pathogen infections.

All classes of proteases are involved in PCD, including cysteine proteases, serine proteases, aspartic proteases and metalloproteases (Beers et al. 2000, 2004; Schaller 2004). In plant PCD, special functions are described for vacuolar proteases (Müntz 2007; Hara-Nishimura and Hatsugai 2011), metacaspases (Tsiatsiani et al. 2011; Lam and delPozo 2000; Xu and Zhang 2009) or subtilisin-like proteases (Vartapetian et al. 2011). It is not clear, however, how these proteases orchestrate PCD and if there is functional redundancy between the different gene families.

Specific for plant PCD is a unique group of papain-type cysteine endopeptidases (CysEPs) characterized by a C-terminal KDEL endoplasmic reticulum (ER) retention signal (KDEL CysEPs) with RcCysEP from castor bean (*Ricinus communis*) as the founding member (Schmid et al. 1998). KDEL CysEPs are not present in mammals or fungi, but are ubiquitous in plants. KDEL CysEPs have been cloned and sequenced from 25 plant species so far; a high degree of homology between the deduced amino acid sequences is apparent; a phylogenetic tree discloses distinct groups among the monocots, dicots and gymnosperms (Hierl et al. 2012).

Castor beans store fat and proteins in a living endosperm, which is laterally attached to the cotyledons. When the mobilization of the storage material is completed after germination, the desiccated endosperm abscises and represents a senescing tissue. Senescing endosperm tissue from castor bean contains a spherical organelle surrounded by a single ribosome-studded membrane with a diameter averaging 1 μm. This organelle was discovered in ultrastructural and cytochemical studies independently by two groups in 1970. It was called “dilated cisternae”, since it seemed to develop from the ER (Vigil 1970), or “ricinosome”, since it was found only in castor bean at that time (Mollenhauer and Totten 1970). The ricinosomes were “re-discovered” with the identification of their marker enzyme, the KDEL-tailed cysteine endopeptidase (Schmid et al. 1998). In *R. communis* the KDEL CysEP and ricinosomes were found not only in the senescing endosperm of germinating seeds (Schmid et al. 1999, 2001), but also in the nucellus of maturing seeds, where the endosperm expands at the expense of the nucellus cells (Greenwood et al. 2005). Crushed cell layers are left behind in the endosperm cells in germinating castor bean seedlings (Schmid et al. 1999, 2001) or in the nucellus cells in maturing castor bean seeds (Greenwood et al. 2005), since there is no need for a photosynthetic plant to recycle the carbon of these cell walls to the surviving parts of the plant. Ricinosomes have been identified by immuno-electron-microscopy in other collapsing tissues undergoing PCD: in senescing flower petals of *Hemerocallis* (Schmid et al. 1999), in the hypogeous cotyledons of *Vicia sativa* (Becker et al. 1997), the unpollinated ovaries of *Pisum sativum* (Cercos et al. 1999), in both developing and dehiscing tomato anthers (Senatore et al. 2009) and in endosperm cells of imbibed tomato seeds (*Solanum lycopersicum*) (Trobacher et al. 2013). The accumulation of KDEL CysEP and the appearance of ricinosomes seem to act as very early predictors of PCD.

KDEL-tailed protease-accumulating vesicles (KDEL vesicles, KVs) in germinating mung bean (*Vigna* *mungo*) cotyledons are similar to ricinosomes in that they accumulate the KDEL-tailed cysteine protease SH-EP (Toyooka et al. 2000). In contrast to ricinosomes, immunocytochemistry identified KDEL vesicles to transport large amounts of SH-EP from the endoplasmic reticulum to protein storage vacuoles. The mass transport of the proteinase by ER-derived KDEL vesicles is thus involved in the protein mobilization of plants (Toyooka et al. 2000; Okamoto et al. 2003).

RcCysEP is synthesized as a pre-pro-enzyme and is co-translationally transferred into the ER, where the pre-sequence is removed. The pro-enzyme is transported from the ER to the cytoplasm of the senescing cells in the form of ER-derived ribosome studded ricinosomes. The final stage of PCD is characterized by destruction of the vacuole integrity, consequent acidification of the cytoplasm and disruption of the ricinosomes, which release the mature RcCysEP. The N-terminal pro-peptide and the C-terminal KDEL-motif are cleaved off, and the activated RcCysEP degrades the cytosolic macromolecules for recycling to the surviving parts of the plant (Schmid et al. 1998, 1999, 2001). RcCysEP exhibits a characteristic and unusual broad substrate specificity. The cleavage site ↓ within a substrate is denoted as P2-P1-↓-P1′-P2′. RcCysEP has a clear preference for neutral amino acids with large aliphatic and non-polar (Leu, Val, Met) or aromatic (Phe, Tyr, Trp) side-chains in the P2 position and no clear preference in the P1 position, as is typical for papain-type CysEPs. RcCysEP accepts proline in the P1 and P1′ positions (Than et al. 2004), which is highly unusual among endopeptidases (Cunningham and O'Connor 1997; Simpson 2001). RcCysEP can therefore digest extensin with its ability to accept glycosylated hydroxy-proline near the cleavage site (Helm et al. 2008). Extensin build the basic scaffold of the cell wall (Cannon et al. 2008), and thus KDEL CysEPs might support final cell collapse. Crystallization of the purified mature RcCysEP revealed that castor bean CysEP folds into two distinct domains of roughly equal size, as is usual for papain-like CysEPs. The RcCysEP folding is also very similar to the proline-specific cysteine peptidase from ginger (*Zingiber officinale*). The active site cleft of RcCysEP, however, is wider than in both the ginger protease and papain (Than et al. 2004). The respective amino acids decisive for this generally more open appearance of the active site cleft—besides the amino acids Cys, His, Gln and Asp defining the catalytic pocket—are highly conserved among all known KDEL CysEPs (Hierl et al. 2012). It can be suggested that all KDEL CysEPs share the same broad substrate specificity.

KDEL CysEPs seem to have a dual role: digesting cytoplasmic components in cells of dying tissues for recycling to the surviving parts of the plant or in cells of germinating seedlings for storage mobilization, respectively, and digesting cell wall extensin in the final stage of PCD or in tissue remodeling.

Castor bean CysEP is thus characterized in terms of biochemistry and cell biology. However, without the *R. communis* genome sequence and in the absence of genetics, it is difficult to see how different gene families work together to orchestrate PCD in different tissues in response to a broad range of developmental or environmental cues.

In *Arabidopsis*, on the other hand, the genome sequence and gene families for all proteases are known. *Arabidopsis* encodes three KDEL CysEPs with homology to the RcCysEP, designated AtCEP1 (At5g50260), AtCEP2 (At3g48340) and AtCEP3 (At3g48350) that are expressed in tissues undergoing PCD. Determination of promoter activities using ß-glucuronidase as reporter in *Arabidopsis* transformants elucidated a remarkable tissue- and organ-specificity. *AtCEP1* and *AtCEP3* promoter activities were found in generative tissues at several stages of seed and fruit development. *AtCEP1*, *AtCEP2*, and *AtCEP3* promoter activities were found in vegetative tissue such as *AtCEP1* in the course of lateral root formation, *AtCEP2* in roots within the beginning root cap, and *AtCEP3* at the hypocotyl-root transition zone or in trichomes of leaves (Helm et al. 2008). However, the *Arabidopsis* CEP proteases have not been characterized at a biochemical level, nor have they been localised intracellularly. The storage organelle for AtCEP1, AtCEP2 and AtCEP3, respectively, their release at the “site of action” and their activation modus remained unknown. There is in fact no evidence for the existence of ricinosomes in *Arabidopsis*, in spite of numerous attempts at isolating such organelles from this model plant.

In this study, we analyzed the root cap as a model tissue for PCD and chose AtCEP2, which is specifically expressed in the course of root cap formation. We used translational fusion proteins of AtCEP2 with a three-fold hemaglutinin-tag (3xHA) and the red fluorescent protein mCherry under the control of the endogenous AtCEP2 promoter (P_CEP2_::pre-pro-3xHA-mCherry-AtCEP2-KDEL) transformed into *Arabidopsis* WT plants. We could then isolate the AtCEP2 protein “ex vivo” and characterize its pH dependent activation, its pH-independent activity and its substrate specificity.

The reporter line expressing P_CEP2_::pre-pro-3xHA-mCherry-AtCEP2-KDEL in a WT background was crossed with the ER membrane marker line expressing a translational fusion protein of green fluorescent protein (GFP) with a ER membrane protein (Cutler et al. 2000) in order to visualize ER-derived subcellular structures functioning as the AtCEP2 storage organelle. We demonstrated AtCEP2 expression in the PCD of root cap formation and identified organelles surrounded by ER-derived membranes as the AtCEP2 storage organelles in young seedlings. Furthermore, we transformed the non-functional reporter protein without the protease subunit, that is, mCherry with the necessary N-terminal and C-terminal targeting signals under the control of the endogenous AtCEP2 promoter (P_CEP2_::pre-pro-3xHA-mCherry-KDEL) into the *atcep2* knockout mutant line in order to facilitate analysis of the *atcep2* knockout mutant phenotype.

## Methods

### Generation of reporter lines expressing pre-pro-3xHA-mCherry-AtCEP2-KDEL, GFP fused to an ER membrane protein in the *Arabidopsis* Col0 WT background, and pre-pro-3xHA-mCherry-KDEL under control of the endogenous AtCEP2 promoter in the *atcep2* knockout mutant

**Fig. 1:**
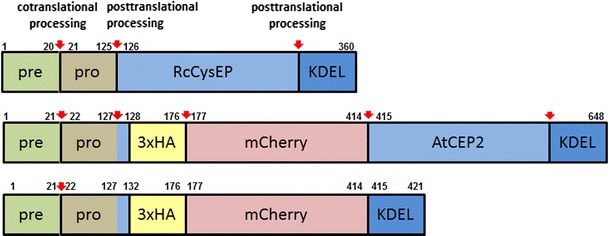
Schematic representation of the fusion proteins pre-pro-3xHA-mCherry-AtCEP2-KDEL and pre-pro-3xHA-mCherry-KDEL expressed under the control of the endogenous AtCEP2 promoter. Pre-pro-RcCysEP is shown for comparison

For the cloning strategy of the fusion gene coding for pre-pro-3xHA-mCherry-AtCEP2-KDEL under the control of the endogenous promoter of AtCEP2 and the primers used see Figs. 1 and S1. The AtCEP2 promoter comprising approximately 2,000 bp with the adjacent 5′UTR and the coding region for the pre-pro-sequence were amplified from WT (Col0) genomic DNA isolated by cetyl-trimethyl-ammonium bromide (CTAB) extraction (Murray and Thompson 1980). The 3xHA tag was amplified from pNIGEL18 and mCherry was amplified from pNIGEL17 (Geldner et al. 2009). The mature AtCEP2 subunit with the 3′UTR was amplified from WT (Col0) genomic DNA. The resulting PCR products were cloned into pGREEN conferring hygromycin resistance (Hellens et al. 2000; www.ac.uk). The final plasmid construct was sequenced and transformed into *Agrobacterium tumefaciens* (pGV3101) by electroporation. Flowers from WT *Arabidopsis* ecotype Columbia (Col0) plants were transformed by floral dipping (Clough and Bent 1998). Eight different homozygous transformants were screened for high expression of the fusion protein by CLSM and three were chosen for further analysis.

The reporter line expressing pre-pro-3xHA-mCherry-AtCEP2-KDEL under the control of the endogenous AtCEP2 promoter was crossed with the GFP-ER membrane protein marker line (Cutler et al. 2000) for determining the subcellular localization of AtCEP2.

The construct P_CEP2_::pre-pro-3xHA-mCherry-KDEL as a non-functional reporter protein lacking the mature AtCEP2 protease subunit was obtained in an analogous manner. It comprised the endogenous AtCEP2 promoter, the 5′UTR and the coding regions for the N-terminal pre-pro-peptide, for the 3xHA tag and mCherry and for the nine C-terminal amino acids of the mature AtCEP2 subunit, including the ER retention signal KDEL (for cloning strategy and primers used see Fig. 1 and S2). The resulting PCR products were cloned into pGREEN (Hellens et al. 2000). The final plasmid construct was sequenced and transformed into *Agrobacterium tumefaciens* (pGV3101) by electroporation. Flowers of *A. thaliana* ecotype Columbia (Col0) plants were transformed by floral dipping. Eight different homozygous transformants were chosen and screened for high expression of the fusion protein by CLSM. The line exhibiting the highest expression level was chosen for crossing with homozygous *cep2* knockout mutant plants.

*atep2* knockout mutant plants (Cold Spring Harbour ET 6591) exhibit the ecotype Landsberg erecta (La-er) background and carry the T-DNA insertion conferring Km resistance approximately in the middle of the first exon (207 bases downstream of the start codon). Homozygous *atcep2* knockout plants were back crossed two times with WT (Col0) plants that were transformed with the non-functional reporter construct P_CEP2_::pre-pro-3xHA-mCherry-KDEL in order to remove unwanted secondary mutations in the *atcep2* knockout mutant line, to replace the La-er background by the Col0 background and to introduce the reporter construct without the protease mature subunit. Finally transformants homozygous for the T-DNA insertion in the first exon of AtCEP2—as confirmed by kanamycin resistance and PCR—in the Col0 background that were expressing the non-functional reporter protein pre-pro-3xHA-mCherry-KDEL—as confirmed by hygromycin resistance and analysis at the CLSM—were obtained.

### Generation of pro-AtCEP2 specific antibodies

Given the large degree of homology between the three members of the AtCEP gene family, polyclonal anti-peptide antibodies were raised that recognized individual family members (Eurogentec, Belgium). Two peptides for AtCEP1, AtCEP2 and AtCEP3 that were specific for each family member were chosen (Figure S3A). An additional Cys at the N-terminus of the peptides was added for coupling to the carrier protein hemocyanin. A mix of the coupled peptides AtCEP2-I (C-QGPKRGSKQFMYDHE) and AtCEP2-II (C-IKLSSSNPTPKDGDV) was used for immunization of two rabbits. Finally, affinity purification of the antisera of the two rabbits against each of the peptides separately was carried out (Eurogentec, Belgium). The AtCEP2-I sequence is located within the pro-peptide and the AtCEP2-II sequence comprises the 15 amino acids upstream of the KDEL-signal. The pro-peptide and the carboxy-terminal ten amino acids including the KDEL-signal are both cleaved off during maturation so the respective antibodies are expected to recognize the pro-form of AtCEP2 but not the mature protein. Anti-peptide antibodies specific for pro-AtCEP1 and pro-AtCEP3 were raised for control. The following peptides were chosen: AtCEP1-I (C-IRMQRGIRHKEGLC), AtCEP1-II (C-LKNSNTNPSRLSLD), AtCEP3-I (C-KTEETYPYDSSDVQFC) and AtCEP3-II (C-TKLSSTPSTHESVVRDDV). The specificity of the anti-peptide antibodies was tested by western blot analysis of pro-AtCEP1, pro-AtCEP2 and pro-AtCEP3 expressed in *E. coli* (Figure S3B). The respective cDNA-clones were used as templates for amplifying and cloning the pro-enzymes of AtCEP1 (RIKEN pda 08738), AtCEP2 (EST clone 99B11) and AtCEP3 (RIKEN pda 12055). Primers for amplification: AtCEP1 (CEP1-NdeI sense 5′ GCAGC CATATG TTA GAT TTC CAT AAC AAA GAT GTG G 3′; CEP1-SalI anti-sense 5′ AGCTT GTCGAC TTA GAG TTC ATC CTT AAG CGA GTC C 3′); AtCEP2 (CEP2-NdeI sense 5′ GCAGC CATATG TTC GAT TAC GAC GAC AAG GAA ATA G 3′; CEP2-SalI anti-sense 5′ AGCTT GTCGAC CTA GAG CTC ATC TTT GAC ATC ACC G 3′); AtCEP3 (CEP3-NdeI sense 5′ GCAGC CATATG TTC GAT TTC GAC GAA AAA GAA TTA GAA ACC 3′; CEP3-SalI anti-sense 5′ AGCTT GTCGAC CTA GAG CTC GTC TTT AAC ATC ATC 3′). The PCR products were sequenced, cloned into pET28 for addition of the N-terminal His-tag and expressed in *E.coli* (Rosetta2, Novagen). Protein extracts from *E. coli* expressing AtCEP1, AtCEP2 or AtCEP3 were used for western blot analysis in order to test the specificity of the anti-peptide antibodies. Castor bean ricinosomes containing RcCysEP (Schmid et al. 2001) and antibodies specific for RcCysEP (Gietl et al. 1997; Schmid et al. 1998) were used for control.

### Generation of antibodies recognizing the mature AtCEP2 subunit

Polyclonal antibodies were raised against the mature AtCEP1 subunit with the expectation that these antibodies would recognize AtCEP1, AtCEP2 and AtCEP3. The *N*-terminal 10 amino acids and the *C*-terminal 13 amino acids of the AtCEP1 mature subunit were omitted in order to obtain antibodies against the mature core sequence of the AtCEP family (Figure S4A). The DNA sequence was cloned into pET24a between the NdeI and XhoI restriction sites and transformed into Rosetta 2 (DE3)pLysS competent cells (Novagen) for expression. The AtCEP1 protein was purified from inclusion bodies by His-tag affinity chromatography using Talon-Metal-Affinity-Resin (Clontech) followed by SDS-PAGE and Coomassie blue staining. The blue stained gel piece was cut out for immunization of rabbits (Davids Biotechnology, Regensburg, Germany). The *N*-terminal amino acid sequence of the AtCEP1 subunit was determined. The mature AtCEP2 subunit was overexpressed and purified in a similar manner (Figure S4A) Cross-reactivity of the antibodies directed against the mature AtCEP1 subunit was tested by western blot analysis of the mature AtCEP1 and AtCEP2 subunit expressed in *E. coli* (Figure S4B).

### Immunoprecipitation of pro-3xHA-mcherry-AtCEP2-KDEL with anti-HA affinity matrix

A protein extract was prepared from approximately 1,500 seven-day-old seedlings (1 g fresh weight). The plant material was ground with mortar and pestle under liquid N_2_ followed by the addition of suspension buffer (50 mM phosphate, 100 mM NaCl, 10 % glycerol pH 7.5; 1 ml/g fresh weight). The suspension was centrifuged (Eppendorff centrifuge, 14,000 rpm, 10 min). The clear supernatant was incubated with 100 μl anti-HA affinity matrix (anti-HA beads; Roche) while gently shaking for 2 h at 8 °C. The anti-HA beads were washed three times with suspension buffer pH 7.5 followed by incubation with assay buffer.

### Analysis of pH-dependent activation, pH-independent activity and cleavage specificity of AtCEP2

**Fig. 2:**
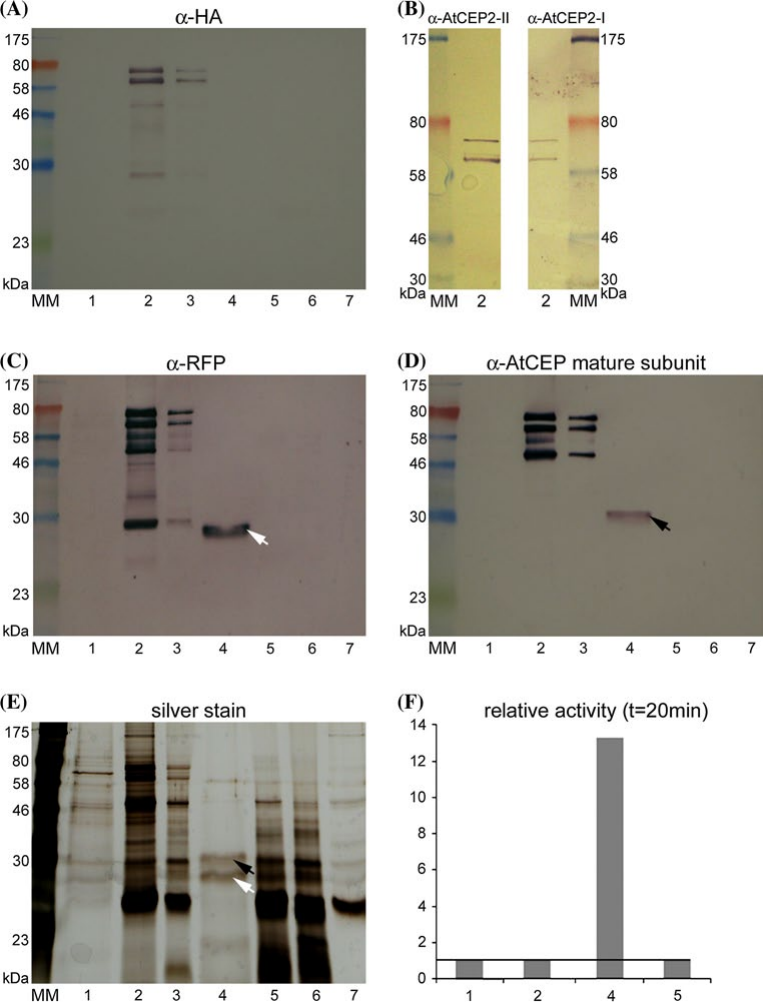
Maturation of pro-3xHA-mCherry-AtCEP2-KDEL by acidification leads to removal of the pro-sequence, cleavage between mCherry and AtCEP2 and release of the enzymatically active AtCEP2 mature subunit. pro-3xHA-mCherry-AtCEP2-KDEL was immunoprecipitated from protein extracts of 7 days old seedlings with anti-HA affinity matrix (anti-HA beads). The anti-HA beads with the pro-3xHA-mcherry-AtCEP2-KDEL attached were incubated with assay buffer pH 7.5 (*lanes* 1–3) or pH 4.0 (*lanes* 4–6), respectively, for 30 min at 25 °C. Incubation of anti-HA beads with buffer instead of plant extract was used as control (*lane* 7). The supernatant (*Lanes* 1 and 4) was separated from the beads; the beads were incubated for 10 min/95 °C followed by separation of this supernatant (*Lanes* 2 and 4) and the now “empty beads” (*Lanes* 3 and 6). The probes (1, 2, 4 and 5) were analyzed directly by measurement of enzymatic activity (**F**). All the probes were analyzed by SDS-PAGE followed by western blot analysis (**A**–**D**) or silver staining (**E**)

**A**nti-HA beads bound to pro-3xHA-mCherry-AtCEP2-KDEL were incubated with 200 μl assay buffer pH 7.5 (100 mM phosphate, 2 mM cysteine, 2 mM DTT, 0.8 %Brij35) or pH 4.0 (100 mM Na acetate, 2 mM cysteine, 2 mM DTT, 0.08 % Brij35) for 30 min at 25 °C. After centrifugation, supernatant and beads were analysed for enzymatic activity with the fluorescence–quenched peptide CBZ-Phe-Arg-AMC (10 mM stock solution in 100 % dimethylformamide, diluted 1:100 for assay) as a substrate for papain-type peptidases; linear kinetics was obtained (Fig. 2F).

Finally, supernatant and anti-HA beads were analysed by SDS-PAGE followed by western blot analysis with anti-HA antibodies (Roche), with anti-RFP antibodies (directed against mCherry; Medical & Biological Laboratories, Japan), with anti-AtCEP2 peptide antibodies detecting pro-3xHA-mCherry-AtCEP2-KDEL and with anti-AtCEP antibodies detecting the mature AtCEP2 subunit or by silver staining: The supernatant separated from the beads (Fig. 2, lanes 1 and 4) was mixed with loading dye (60 mM Tris–HCl pH 6.6, 5 % glycerine, 1.5 % SDS, 1.5 % ß-ME, 0.1 % bromophenol blue final concentration) and incubated for 10 min/95 °C before loading; the beads were mixed with loading dye and incubated for 10 min/95 °C followed by separation of this supernatant (Fig. 2, lanes 2 and 5) and the now “washed” beads itself by centrifugation; the “washed” beads were finally mixed with loading dye (Fig. 2, lanes 3 and 6) and analyzed by SDS-PAGE. As control, the anti-HA beads were treated in a similar manner without adding plant material; the finally “washed” beads were analysed by SDS-PAGE (Fig. 2, lane 7) in order to control the unspecific signals obtained by the anti-HA beads.

**Fig. 3:**
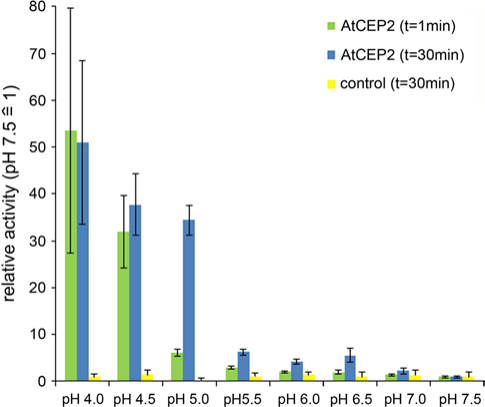
pH dependence of activation. pro-3xHA-mCherry-AtCEP2-KDEL was immunoprecipitated with anti-HA affinity matrix and incubated at different pH values for 1 min or 30 min, respectively. Subsequently, the relative enzymatic activity was measured with the fluorescence quenched substrate CBZ-Phe-Arg-AMC. A linear kinetic was obtained. Control: immunoprecipitation with anti-HA affinity matrix from untransformed WT plants. Acetate buffer pH 4.0, 4.5, 5.0; phosphate buffer pH 5.5, 6.0, 6.5, 7.0, 7.5, 8.0. Standard deviation: n = 3

pH dependent activation: anti-HA beads with the pro-3xHA-mCherry-AtCEP2-KDEL were incubated with assay buffer at different pH values (100 mM Na acetate, 2 mM cysteine, 2 mM DTT, 0.08 % Brij35 at pH 4.0, pH 4.5, pH 5.0; 100 mM phosphate, 2 mM cysteine, 2 mM DTT, 0.08 % Brij35 at pH 5.5, pH 6.0, pH 6.5, pH 7.0, pH 7.5, pH 8.0) for 1 min or 30 min, and analysed for enzymatic activity with CBZ-Phe-Arg-AMC as substrate (Fig. 3).

**Fig. 4:**
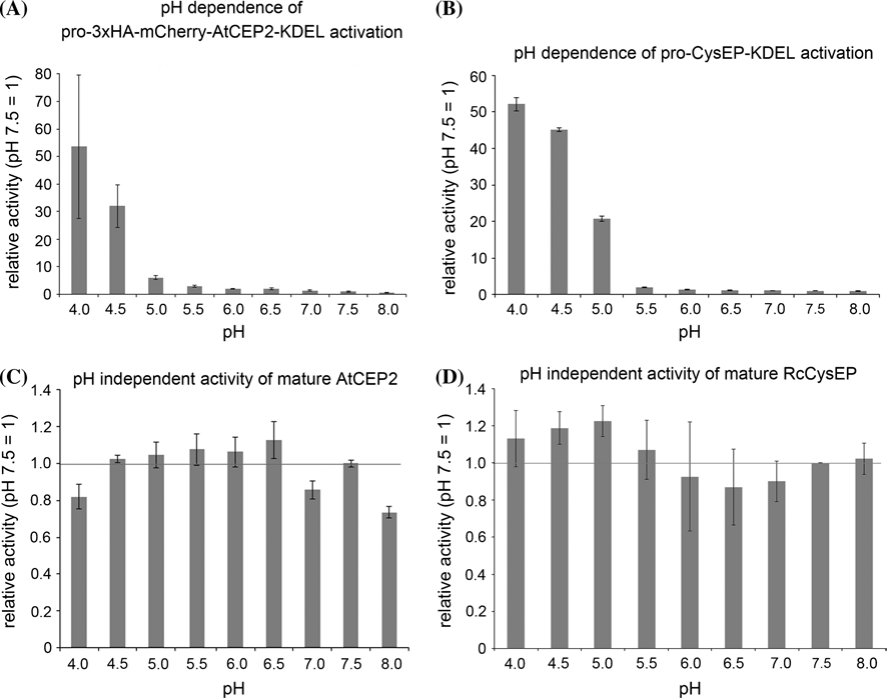
pro-3xHA-mCherry-AtCEP2-KDEL and pro-CysEP-KDEL exhibit similar pH dependence of activation (**A**, **B**) and pH independence of activity (**C**, **D**). **A**, **B** Activation at different pH values for 1 min followed by measurement of enzymatic activity for 20 min with the fluorescence quenched substrate CBZ-Phe-Arg-AMC. Acetate buffer pH 4.0, 4.5, 5.0; phosphate buffer pH 5.5, 6.0, 6.5, 7.0, 7.5, 8.0. **C**, **D** Activation for 30 min at pH 4.5 in 10 μl buffer followed by addition of 190 μl buffer of the indicated pH and measurement of enzymatic activity for 20 min. A linear kinetic was obtained in all experiments. Standard deviation: n = 3

pH independent activity: anti-HA beads with the pro-3xHA-mCherry-AtCEP2-KDEL were incubated with 10 μl activation buffer (50 mM Na acetate, 2 mM cysteine, 2 mM DTT, 0.08 % Brij35 pH 4.5) for 30 min at 25 °C; 190 μl assay buffer of different pH values were added (100 mM Na acetate, 2 mM cysteine, 2 mM DTT, 0.08 % Brij35 at pH 4.0, pH 4.5, pH 5.0; 100 mM phosphate, 2 mM cysteine, 2 mM DTT, 0.08 % Brij35 at pH 5.5, pH 6.0, pH 6.5, pH 7.0, pH 7.5, pH 8.0) and enzymatic activity was measured for 20 min. For comparison of AtCEP2 with RcCysEP, purified ricinosomes containing the pro-CysEP (Schmid et al. 2001) were used instead of immunoprecipitated pro-3xHA-mCherry-AtCEP2-KDEL (Fig. 4).

Cleavage motifs of AtCEP2 were analysed by digestion of bovine beta-casein purchased from Sigma (C-6905). Anti-HA beads with the pro-3xHA-mCherry-AtCEP2-KDEL were incubated with buffer at pH 4.5 for 30 min at 25 °C in order to obtain the mature, active AtCEP2. Beta-casein was added (0.13 % final concentration) and incubation at 25 °C was continued. Aliquots were taken after 1 min, 10 min, 30 min, 4 h and 24 h and analysed by 17.5 % SDS-PAGE followed by Coomassie-staining. Digests were stopped by freezing in liquid nitrogen. The same time points were chosen for further analysis: The beta-casein peptides obtained by digestion with AtCEP2 for 1, 10, 30 min, 4 and 24 h were separated by reversed-phase HPLC prior to N-terminal sequencing and MALDI analysis as previously described (Than et al. 2004).

### Confocal laser scanning microscopy

Confocal laser scanning microscopy (Fluoview FV 1,000, Olympus, Japan) was performed using excitation at 561 nm and emission detection between 600 and 630 nm for mCherry and excitation at 480 nm and emission detection between 510 and 530 nm for GFP. Single pictures or stacks of pictures with 0.5 μm step size at higher resolution and 2.5 μm step size at lower resolution were made. SYTO^®^ 13 Green-Fluorescent Nucleic Acid Stain (Invitrogen) was used for staining of nuclei in root cap cells: seedlings were immersed for 3 min in SYTO^®^ 13 (5 μM final concentration in buffer 20 mM Tris, 45 mM NaCl pH 7.4) followed by CLSM.

## Results

### Generation of reporter lines expressing pre-pro-3xHA-mCherry-AtCEP2-KDEL, GFP fused to an ER membrane protein in the *Arabidopsis* Col0 WT background, and pre-pro-3xHA-mCherry-KDEL under control of the endogenous AtCEP2 promoter in the *atcep2* knockout mutant

We constructed the gene for fusion proteins including mCherry with and without the mature AtCEP2 subunit (Fig. 1). The fusion genes were cloned under the control of the endogenous AtCEP2 promoter thus obtaining P_CEP2_::pre-pro-3xHA-mCherry-AtCEP2-KDEL transformed into *Arabidopsis* Col0 WT plants and the non-functional reporter P_CEP2_::pre-pro-3xHA-mCherry-KDEL transformed into *cep2* knock out mutant plants (Fig. 1; Figures S1 and S2).

The sequence approximately 2,000 bp upstream of the start Met, that were previously shown to confer tissue specific expression (Helm et al. 2008), were used as the AtCEP2 promoter region. We placed the first three amino acids Leu-Pro-Ser of the mature subunit C-terminal to the pro-sequence in front of the 3xHA tag in order to ensure processing of the pro-peptide during maturation of AtCEP2. The junction between mCherry and the mature AtCEP2 subunit, on the other hand, exhibited only the “half” of the recognition site for processing to the mature subunit in order to obtain the mCherry-mature AtCEP2 protease subunit fusion protein after maturation (Fig. 1; Figure S1). The final plasmid construct was sequenced and transformed into *Agrobacterium tumefaciens* for subsequent transformation of WT (Col0) plants.

The resulting reporter line expressing pre-pro-3xHA-mCherry-AtCEP2-KDEL under the control of the endogenous AtCEP2 promoter was crossed with the *Arabidopsis* reporter line expressing a fusion protein between GFP and an ER membrane protein (Cutler et al. 2000) in order to visualize the subcellular structure that functions as the storage organelle for AtCEP2.

The construct P_CEP2_::pre-pro-3xHA-mCherry-KDEL as a non-functional reporter protein without the mature AtCEP2 protease subunit was obtained in an analogous manner. It comprised the endogenous AtCEP2 promoter, the 5′UTR and the coding regions for the N-terminal pre-pro-peptide, for the 3xHA tag and mCherry and for the C-terminal nine amino acids of the mature AtCEP2 subunit including the ER retention signal KDEL (Fig. 1; Figure S2). It was transferred into *cep2* knockout mutant plants.

### Maturation of pro-3xHA-mCherry-AtCEP2-KDEL

Analysis of the tissue- and organ-specific activities of the three KDEL CysEP promoters in *Arabidopsis* transformants revealed significant *AtCEP2* promoter activity in young seedlings (Helm et al. 2008). *AtCEP2* promoter activity was especially striking in root tips, and probably in the calyptra and the root elongation zone of the root.

A protein extract was prepared from seven-day-old seedlings. The fusion protein pro-3xHA-mCherry-AtCEP2-KDEL was immunoprecipitated with anti-HA affinity matrix (anti-HA beads) at pH 7.5. The beads were washed with buffer at pH 7.5 and subsequently incubated with 200 μl assay buffer at pH 7.5 (Fig. 2A–E, lanes 1–3) or pH 4.0 (Fig. 2A–E, lanes 4–6). As a control, anti-HA beads were incubated with buffer instead of protein extract (Fig. 2A–E, lane 7). After separation of supernatant and beads by centrifugation, supernatant and beads were analysed for enzymatic activity with the fluorescence–quenched peptide CBZ-Phe-Arg-AMC (Fig. 2F). Supernatant and beads were subsequently analysed by SDS-PAGE, followed by western blot analysis with anti-HA antibodies Fig. 2A), with anti-AtCEP2 peptide antibodies detecting pro-3xHA-mCherry-AtCEP2-KDEL (Fig. 2B), with anti-RFP antibodies directed against mCherry (Fig. 2C), or with antibodies recognizing the mature AtCEP2 subunit (Fig. 2D), respectively, and by SDS-PAGE followed by silver staining (Fig. 2E).

Immunprecipitation of pro-3xHA-mCherry-AtCEP2-KDEL with anti-HA beads and incubation at pH 7.5 revealed two distinct protein bands as recognized by anti-HA antibodies (Fig. 2A). Both proteins exhibit molecular masses smaller than the 80 kDa MW marker and represented the pro-3xHA-mCherry-AtCEP2-KDEL protein with a calculated MW of 70.5 kDa. At pH 7.5, these proteins were not released into the supernatant (Fig. 2A, lane 1), but remained attached to the anti-HA beads and had to be solubilized by treatment with loading dye at 95 °C for 10 min (Fig. 2A, lane 2). Both proteins exhibited no enzymatic activity (Figs. 2F, 3) and thus should contain the intact pro-peptide. Both protein bands were recognized by the antiAtCEP2-I pro-peptide antibody (Fig. 2B, right) and by the anti-AtCEP2-II peptide antibody directed against the peptide at the extreme carboxy-terminus (Fig. 2B left and Figure S3), by anti-RFP- (Fig. 2C, lane 2) and by anti-AtCEP-antibodies (Fig. 2D, lane 2 and Figure S4).

Immunoprecipitation of pro-3xHA-mCherry-AtCEP2-KDEL with anti-HA beads, followed by incubation at pH 7.5 and decoration with anti-HA antibodies revealed two more relatively faint protein bands: one with a MW < 58 kDa that probably represents 3-HA-mCherry-AtCEP2 with a calculated MW of 57.2 kDa, and another that is probably 3xHA-mCherry lacking the pro-peptide and the AtCEP2 mature (Fig. 2A–D, lane 2). The AtCEP2 mature subunit adjacent to 3xHA-mCherry seems to be below the level of detection using antibodies or by enzymatic activity (Fig. 2D, F, lane 2). A small amount of all three HA-tagged proteins could not be solubilised and remained attached to the anti-HA beads (Fig. 2, lane 3).

Immunoprecipitation of pro-3xHA-mCherry-AtCEP2-KDEL with anti-HA beads and incubation at pH 4.0 resulted in maturation by self-cleavage and in release from the anti-HA beads to the supernatant (Fig. 2A, C and D: lane 4). Most interesting, not only were the KDEL-motif and pro-peptide removed, but mCherry (Fig. 2, lane 4, white arrow) and the mature AtCEP2 subunit (Fig. 2, lane 4, black arrow) were separated. The AtCEP2 mature protease in the supernatant (Fig. 2D, lane 4) was enzymatically active (Fig. 2F, lane 4). Maturation and cleavage between mCherry and AtCEP2 mature subunit at pH 4.0 was confirmed by SDS-PAGE and silver staining (Fig. 2E, lane 4). pH-dependent activation could also be followed by CLSM: incubation of the anti-HA affinity matrix containing the immunoprecipitated pro-3xHA-mCherry-AtCEP2-KDEL with assay buffer at pH 7.5 resulted in a “stably localized” mCherry signal around the beads, whereas incubation with assay buffer pH 4.0 resulted in a diffuse mCherry signal spreading away from the beads. Our AtCEP2 reporter fusion construct thus represents a protein functional in vivo.

The pH dependence of AtCEP2 activation was analysed in more detail by incubating the anti-HA affinity matrix containing the immunoprecipitated pro-3xHA-mCherry-CEP2-KDEL at pHs between pH 7.5 and 4.0. The relative activity with the fluorescence quenched peptide CBZ-Phe-Arg-AMC for 20 min exhibited a linear progress (Fig. 3). As a control we used anti-HA affinity matrix incubated for immunoprecipitation with protein extract from untransformed WT plants not expressing the HA tagged AtCEP2 fusion protein (Fig. 3). Between pH 5.5 and pH 7.5, only a weak activation close to the back ground could be observed. The turning-point was observed at pH 5.0, where after 1 min a 5-fold increase of relative fluorescence units was reached and after 30 min activation time, a 35-fold increase of relative fluorescence units was reached. At pH 4.5 and at more acidic pH values the full activity of the mature AtCEP2 was reached after 1 min activation time (Figs. 3, 4A).

When activated (incubation time at pH 4.5 for 30 min), the mature AtCEP2 exhibited a pH independent activity reaching similar relative fluorescence units within 20 min measurement at all pH values between pH 4.0 and pH 8.0 (Fig. 4C).

This pH-dependent activation and pH-independent activity of AtCEP2 was compared with that of RcCysEP from isolated ricinosomes. RcCysEP had its “turning-point” at pH 5.0 and reached full enzymatic activity within 1 min at pH 4.5 and 4.0 (Fig. 4B). Mature castor bean CysEP exhibited a pH-independent activity similar to AtCEP2: acidification of isolated ricinosomes for 30 min at pH 4.5 revealed the mature CysEP that had a similar enzymatic activity at all pH values between pH 4.0 and pH 8.0 (Fig. 4D).

The pro-enzymes of AtCEP2 and RcCysEP isolated “ex vivo” either by immunoprecipitation with anti-HA-affinity matrix or by purification of ricinosomes, exhibit similar biochemical properties in that acidification at pH 5.0 and at more acidic pH values leads to self-cleavage of the pro-peptide, and to maturation of the pro-enzyme to the mature and enzymatically active protease.

### AtCEP2 accepts proline near the cleavage site thus exhibiting the broad substrate specificity typical for KDEL-CysEPs

We examined peptide fragments generated by digestion of bovine milk beta-casein with AtCEP2. Peptides were separated by reversed-phase HPLC prior to N-terminal sequencing and MALDI-TOF analysis. The sequences of the 80 peptides identified revealing 57 different cleavage sites are listed in Table S1. An additional six cleavage sites were identified by N-terminal amino acid sequencing (EELN…., ELNV…, IEKF…, KFQS…, NSLP…, VPPF…). Figure S5 shows the beta-casein sequence with the identified cleavage sites marked by arrows.

Neutral amino acids with large aliphatic and non-polar residues or with aromatic residues were preferentially found at the P2 and P2′positions (Figure S5). A clear preference at the P1 and P1′ positions was not apparent (Figure S5). Of particular interest was the acceptance of Pro at P2, P1, P1′ P2′ (Figure S5). AtCEP2 can thus cleave at both the N- and C-terminal side of proline. This highly unusual proline-specific cleavage seems to be a characteristic feature of KDEL CysEPs. The biochemical data therefore confirmed that the translational fusion protein pre-pro-3xHA-mCherry-AtCEP2-KDEL is functional.

### AtCEP2 is expressed in the final stage of root cap formation and in root elongation

Analysis of seedlings between 2 and 35 days old expressing pre-pro-3xHA-mCherry-AtCEP2-KDEL in the WT background by western blot analysis of protein extracts with anti-HA-tag antibodies and by CLSM indicated that AtCEP2 was expressed from the beginning of seedling growth in root caps as soon as the root tips emerged.

**Fig. 5:**
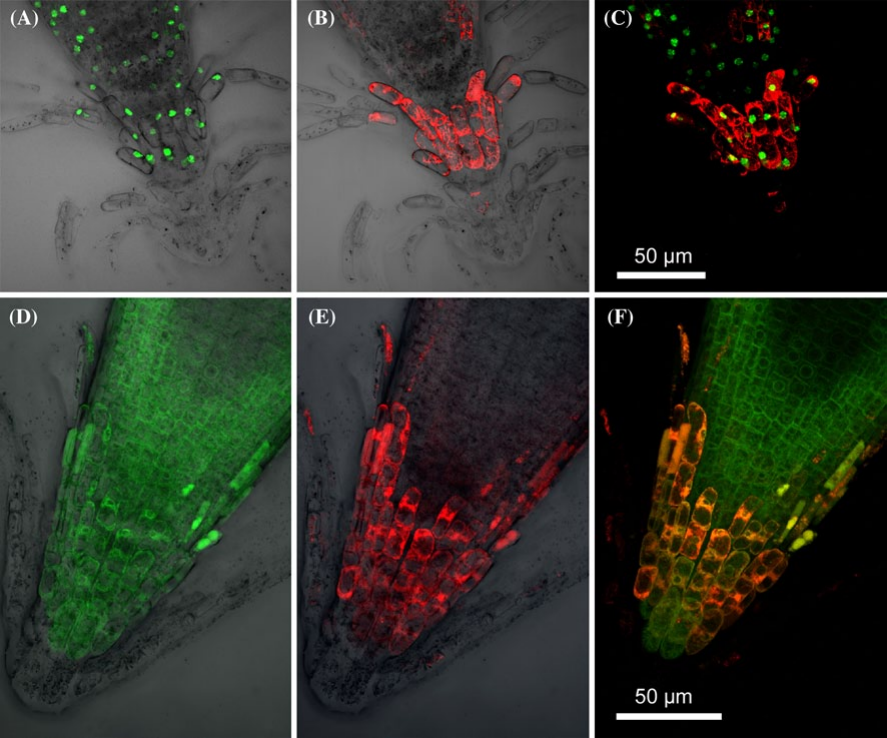
Localization of the mCherry-AtCEP2 reporter protein and the nuclei stained with the DNA-stain SYTO^®^ 13 in the root cap (**A**–**C**) and localization of the mCherry-AtCEP2 reporter protein and the GFP-ER membrane reporter protein (**D**–**F**). **A** The DNA stain marks the nuclei of the root and the latest root cap. **B** The mCherry-AtCEP2 reporter is most strongly expressed in cells of the latest root cap. **C** Merge of the AtCEP2 reporter and the stained nuclei reveals the strongest expression of the AtCEP2 reporter in the last cells carrying a stainable nucleus indicating the following cells to be dead. **D**–**F** The mCherry-AtCEP2 can be detected in cells of the already released root cap and in root tip cells that are developing into the next root cap cells. Co-expression of mCherry-AtCEP2 and a GFP-ER membrane protein marker (Cutler et al. 2000) reveals a partial co-localization indicating that AtCEP2 might be localized to subdomains of the ER. **A**–**C** Stacks of images were obtained with a step size of 1.0 μm; enlargement 600×. **D**–**F** Stacks of images were obtained with a step size of 0.5 μm; enlargement 600×

**Fig. 6:**
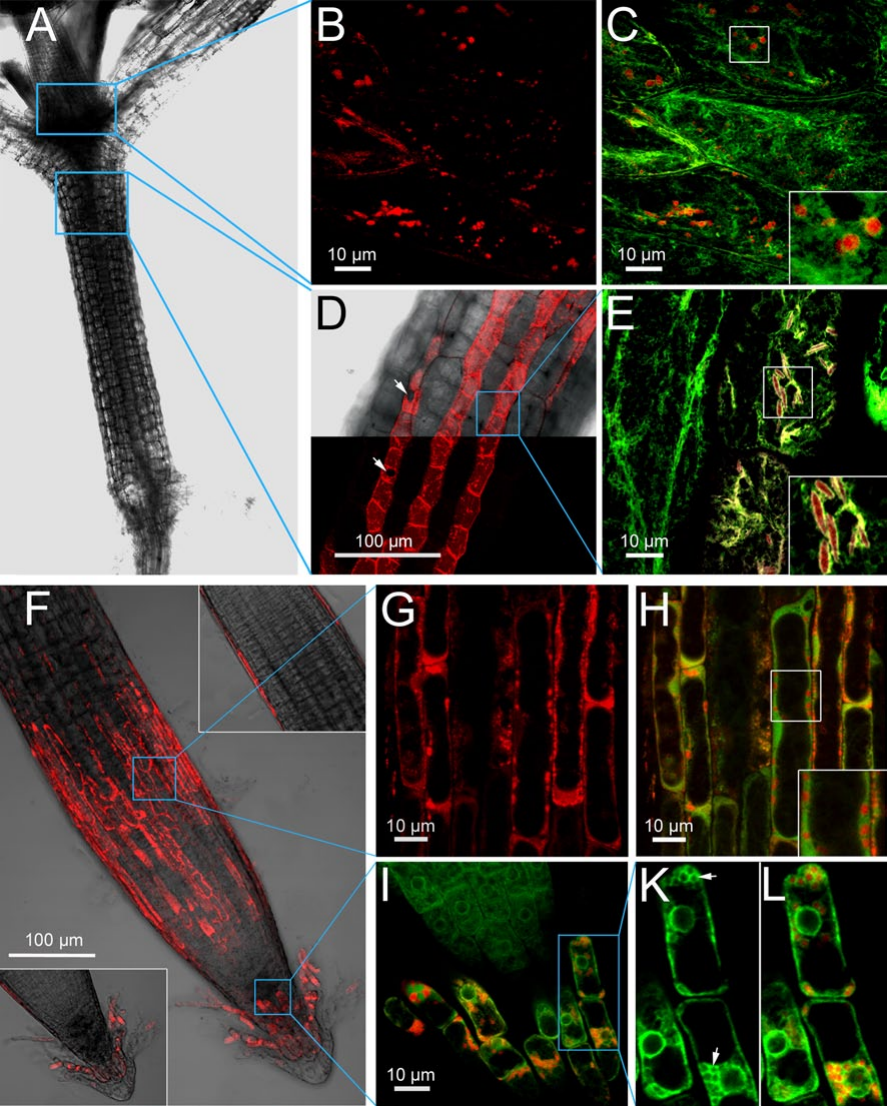
Sub-cellular co-localization of mCherry-AtCEP2 and a GFP-ER membrane protein in epidermis cells at the base of young leafs, in the hypocotyl and in the root cap of *Arabidopsis thaliana* seedlings. **A** Light micrograph (differential interference contrast) of an *Arabidopsis* seedling. *Blue boxes* indicate representative tissues, where the enlarged images (**B**–**E**) can be found. **B**, **C** mCherry-AtCEP2 at the base of young leafs is stored in round, ricinosome-like ER-derived vesicles; **B** *red* channel showing mCherry-AtCEP2; **C** merge of *red* mCherry-AtCEP2 and *green* ER membrane protein. **D** mCherry-AtCEP2 is observed in epidermis cells of the hypocotyl only in cell files including the stomata (*white arrow*); the upper part shows the red channel and differential interference contrast, the lower part shows the *red* channel only. **E** Enlargement of the tissue boxed in (**D**) elucidates the co-localization of mCherry-AtCEP2 and the GFP-ER membrane protein in spindle-shaped ER-derived vesicles resembling ER-bodies. **F** Roots exhibits a distinct and characteristic two band pattern; the upper band seems to mark the root elongation zone, whereas the signal in the root tip is localized in the latest root cap cells. **G**, **H** Co-localisation of mCherry-AtCEP2 with distinct areas of the ER is observed. AtCEP2 is found in round ricinosome-like organelles. **I**, **K**, **L** AtCEP2 can be localized in the latest root cap cells to ER-derived organelles; numerous round organelles resembling ricinosomes (Fig. 6K, *white arrows*) are found that were filled with AtCEP2. **A**, **F** the *blue boxes* indicate representative tissues, where the enlarged images can be found. **C**, **E**, **H** the *white insets* represent enlargements of the *white boxed* areas. **K** and **L** represent enlargements of the tissue boxed in** I**. Stacks of images were obtained. **D**, **F**: Step size 1 μm; *insets* in** F**. single images. **B**, **C**, **E**, **G**, **H**, **I**, **K** step size 0.5 μm

Seven days old seedlings were analysed by CLSM (Fig. 5). The DNA stain SYTO^®^ 13 Green-Fluorescent Nucleic Acid Stain marked the nuclei of the root and the latest root cap (Fig. 5A), whereas the AtCEP2 reporter protein is most strongly expressed in cells of the latest root cap (Fig. 5B). Merging the AtCEP2 reporter and the stained nuclei revealed the greatest expression of the AtCEP2 reporter in the last cells carrying a stainable nucleus, indicating that the oldest cells of the root cap were dead (Fig. 5C). Localization of the red AtCEP2 reporter (Fig. 5E) and the green ER membrane protein reporter (Fig. 5D) in the root tip revealed co-localization of pro-3xHA-mCherry-AtCEP2-KDEL in distinct areas of the ER in the root cap (Fig. 5F). A diffuse labelling could be observed in a few outermost root cap cells, suggesting that their vacuole was disrupted (Fig. 5D–F). Expression of the AtCEP2 reporter protein caused a distinct two band pattern in the root, where the upper band marked the root elongation zone and the lower band represented the signal in the youngest root cap cells of the root tip (Fig. 6F). Single CLSM pictures show the localization of AtCEP2 exclusively in the outermost layer of the root, that is in the latest root cap cells peeling off and in the epidermis of the elongation zone; no AtCEP2 expression was seen in cells inside the root (Fig. 6F, insets). It should be noted that—in contrast to root cap formation—PCD does not occur in the root elongation zone.

In principle, the expression of the *AtCEP’s* in roots is reflected in the “root expression visualizer (www.arexbd.org database)” (Figure S6). However, no probeset ID is available for *AtCEP2* (At3g48340) in the ATH1 datasource. The *AtCEP2* expression pattern has erroneously been attributed to *AtCEP1* (At5g50260, probe ID 248545_at) or AtCEP3 (At3g48350; probe ID_252368_at).

**Fig. 7:**
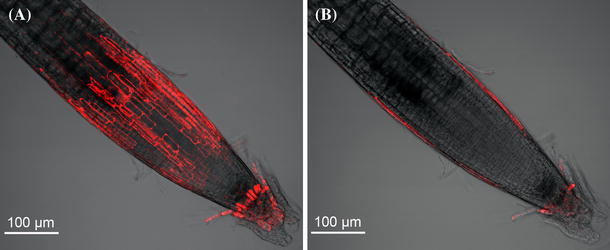
*atcep2* KO mutant plants exhibit no obvious phenotype such as an impairment in root cap formation compared to WT plants. *at*cep2 KO mutant line transformed with the non-functional reporter protein pre-pro-3xHA-mCherry-KDEL. **A** Stacks of images were obtained with a step size of 1 μm. **B** single image

Interestingly, *atcep2* knockout mutant lines exhibited no obvious phenotype such as impairment in root elongation or cap formation (Fig. 7). We analysed the *atcep2* knockout mutant line expressing pre-pro-3xHA-mCherry-KDEL. No difference to WT root cells was observed. Roots exhibited a normal root cap; expression of the non-functional reporter without the AtCEP2 protease subunit revealed the characteristic two band pattern (Fig. 7).

Expression levels of *AtCEP1* and *AtCEP3* were 1.9-fold and 1.8-fold up-regulated in 7 days old seedlings of *atcep2* knock out plants in comparison with those of the wild type (Figure S7). In young seedlings, *AtCEP1* is expressed in the root tip and during lateral root formation and *AtCEP3* is expressed in the hypocotyl-root transition zone and within the main root inside the endodermis (Helm et al. 2008). The slight up-regulation of *AtCEP1* and *AtCEP3* in *atcep2* knock out plants might take place in these tissues without a change of the promoter specificity of these KDEL peptidases.

**Fig. 8:**
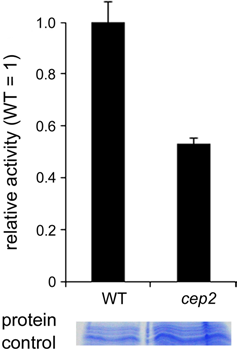
AtCEP2 amounts to more than 40 % of the papain-type protease activity in 7 days old seedlings. Seven days old seedlings from wild type and *atcep2* knock out plants were analyzed for enzymatic activity with the fluorescence–quenched peptide CBZ-Phe-Arg-AMC at pH 4.5 (n = 3). For control of the protein amounts obtained and measured in the protein extract, the protein extracts were compared by SDS-PAGE; a representative area is shown

Comparison of proteolytic activity in a protein extract from wild type plants and from the *atcep2* knockout mutants indicated a reduction of proteolytic activity to <60 % in the *atcep2* knockout mutant plant compared to wild type (Fig. 8). AtCEP2 might therefore account for more than 40 % of the papain-type proteolytic activity in roots.

### AtCEP2 is expressed in young primary leaves and in the hypocotyl

The AtCEP2 reporter protein was also expressed in epidermis cells at the base of young leaves (Fig. 6A–C). AtCEP2 was expressed in the hypocotyl in cells of non-protruding cell files (Fig. 6A, D, E) but not in the stomata itself (Fig. 6D, white arrows).

### AtCEP2 in young seedlings is exclusively expressed in epidermal cells and is localized to ER-derived organelles

Confocal laser scanning microscopy analysis of WT plants co-expressing the red AtCEP2 reporter protein and a green ER membrane protein (Cutler et al. 2000) established storage of AtCEP2 in ER-derived organelles: vesicles filled with the red AtCEP2 reporter are surrounded by a green membrane (Figs. 6C, E, H, K and L). We found two types of storage organelles: round ricinosome-like organelles with their characteristic diameter of approximately 1 μm and ER-bodies with their characteristic 10 μm long spindle-shaped appearance.

Epidermis cells at the base of young leaves exhibited the round ricinosome-like organelles; no ER-bodies were found (Fig. 6B, C).

Epidermis cells of the hypocotyl showed exclusively the spindle-shaped ER-bodies; no ricinosome-like organelles could be detected (Fig. 6D, E). Interestingly, cells in the non-protruding cell files containing the stomata were “full” of ER-bodies that could be seen due to their length at lower magnifications. On the other hand, cells in the protruding cell files where no stomata are formed exhibited no AtCEP2-containing organelles (Fig. 6D).

In cells of the root elongation zone (Fig. 6G, H) and in root cap cells (Fig. 6I–L), co-localization of AtCEP2 with distinct areas of the ER was observed: AtCEP2 can be localized to ricinosome-like organelles, that is, round bodies filled with the red AtCEP2 reporter and surrounded by a green membrane.

A careful examination of the tissues analyzed in the 7 days old seedlings revealed that either ricinosome-like organelles or ER-bodies harbouring mCherry-AtCEP2 were found in the same tissue; both types of organelles labeled with AtCEP2 could not be detected in the same cell or tissue.

## Discussion

We could show that AtCEP2 is expressed in vegetative tissues such as young *A. thaliana* seedlings, that is, in the root cap and root elongation zone, in the non-protruding cell files of the hypocotyl containing stomata (but not in the stomata itself) and at the base of young leaves. We found AtCEP2 exclusively in epidermal cells, where it is stored in round ricinosome-like organelles or in spindle-shaped ER-bodies as the enzymatically inactive pro-form, although very small amounts of the mature subunit seem to present at neutral pH. Acidification of pro-AtCEP2 results in maturation, that is, cleavage of the pro-peptide and also cleavage between mCherry and the AtCEP2 mature protease, indicating that the pre-pro-3xHA-mCherry-AtCEP2-KDEL reporter is a functional construct in vivo. The similarity to RcCysEP suggests that this pH-dependent activation is common to KDEL-CysEPs. A small amount of the mature AtCEP2 protease was also present, consistent with our finding with the RcCysEP (Schmid et al. 1998): the assay at pH7.5 with the radioactively labelled precursor of glyoxysomal malate dehydrogenase (pre-gMDH) as a substrate revealed the pro-form to exhibit a very low proteolytic activity.

The presence of a GFP-tagged ER membrane protein showed that AtCEP2 was localized in two different ER-derived compartments: in spherical 1 μm organelles (similar to ricinosomes or KDEL vesicles) and in 1 × 10 μm spindle-shaped ER-bodies. AtCEP2 was expressed exclusively in epidermal cells: at the base of young leaves, in the hypocotyl, in the root elongation zone and in the root cap of seedlings.

Plants exhibit several organelles for transport of proteins synthesized at the ER to their final destination. In general, proteins for secretion are synthesized at the ER and are modified in the ER lumen or Golgi, respectively, through formation of disulfide bridges and glycosylation before they reach their final destination by vesicle transport. Most transport vesicles from the ER are coat protein II (COPII) vesicles with a diameter of 50 nm. Plants develop various ER-derived structures with a diameter between 200 nm and 1 μm for protein storage (Hara-Nishimura et al. 2004; Herman and Schmid 2004). Monocotyledons produce “protein bodies (PB)” for accumulation of seed storage proteins (Herman and Larkins 1999; von Wettstein 2007). “Precursor-accumulating (PAC) vesicles” in the maturing cotyledons of pumpkin (*Cucurbita* *maxima*) are instrumental in the bulk transport of seed protein precursors (Hara-Nishimura et al. 1998, 2004).

The “ER body” is a unique organelle of Brassicales plants (for a recent review see Yamada et al. 2011). ER bodies are spindle-shaped structures surrounded by a single membrane with attached ribosomes. With a size of 10 μm long and 1 μm wide, they are the largest ER structure in plants. ER bodies can be categorized into two types in *Arabidopsis*: seedling and root ER bodies, and wound-inducible ER bodies. *Arabidopsis* seedlings accumulate ER bodies in the epidermis. ER bodies are found in cotyledons and hypocotyl, where they disappear with the progression of senescence, and in root tissues, where ER bodies are constitutively accumulated; when plants are wounded, the accumulation of ER bodies increases (Matsushima et al. 2002). ER bodies have specific membrane proteins termed “membrane protein of endoplasmic reticulum body 1″ (MEB1) and MEB2 that localize to the ER body membrane but not to the ER network; MEB1 and MEB2 are suggested to be metal transporters since they suppress iron and manganese toxicity upon heterologous expression in yeast (Yamada et al. 2013). Several ß-glucosidases with an ER retention signal were described as the main components in the ER body, which lets suggest that ER bodies are involved in the metabolism of glycosides such as glucosinolates (Matsushima et al. 2003; Yamada et al. 2009). It is suggested that the ER body is involved in defense against metal stress as well as pathogens and herbivores (Yamada et al. 2013).

We found ER bodies harbouring mCherry-AtCEP2 in epidermis cells of the hypocotyl; this might indicate that ER bodies in *Arabidopsis* are storage organelles not only for ß-glucosidases but also for AtCEP2. It is possible that both enzymes may be present at the same time. On the other hand, we found numerous AtCEP2-storing ricinosome-like organelles in roots, but apparently no AtCEP2-storing ER bodies, although ß-glucosidase-accumulating ER bodies should be present in root tissues. Future experiments visualizing ER bodies with a luminal KDEL-tailed GFP should clarify if ricinosomes and ER bodies are simultaneously present, and if AtCEP2 is routed to only one of the organelles, depending on the cell type.

Ricinosomes were established as storage organelle for the pro-form of KDEL-CysEPs in several plants such as castor bean endosperm of germinating seeds and nucellus of maturing seeds (Schmid et al. 1999, 2001; Greenwood et al. 2005) and tomato anthers and imbibed seeds (Senatore et al. 2009, Trobacher et al. 2013); in these tissues, the appearance of ricinosomes and the accumulation of KDEL CysEP are an early indicator for the final stage of developmental PCD. Ricinosome-like organelles have now been found for the first time in *Arabidopsis*. AtCEP2 storing ricinosomes in *Arabidopsis* seedlings seem to be—like ER bodies—exclusively localized in epidermal cells. Interestingly, AtCEP2-storing ricinosome-like organelles were found not only in the root cap as a prominent example for developmental PCD, but mCherry-AtCEP2 containing ricinosome-like vesicles surrounded by the GFP-labeled ER-derived membrane were also present in the root elongation zone, where no PCD is to be expected. KDEL CysEPs are not glycosylated (Than et al. 2004); together with the KDEL endoplasmatic reticulum retention signal, a localization to Golgi bodies or the TGN is not to be suggested.

ER bodies are suggested to be involved in defence against biotic and abiotic stress. A role for KDEL-CysEPs and ricinosomes in pathogen defense, for example against biotrophic fungi, cannot be excluded. Depending on the developmental stage or the necessity for pathogen defense of the plant, ricinosomes could be the storage organelle not only for KDEL CysEPs but also for other, yet unknown enzymes. For example, ricinosomes in the nucellus of young maturing castor bean seeds exhibit a grape-like structure and contain a diverse spectrum of proteins in addition to the KDEL CysEP (Greenwood et al. 2005), whereas the matrix enzymes of ricinosomes in the endosperm of germinating castor bean seeds obviously mainly consist of KDEL-CysEP (Schmid et al. 2001).

An alternative pathway for transfer of proteases from the ER via the Golgi apparatus to vacuoles prior to PCD has been identified in the endothelium cells of maturing *Arabidopsis* seeds (Ondzighi et al. 2008). The endothelium is the layer of cells that surrounds the endosperm and undergoes PCD during embryogenesis. A protein disulfide isomerase with a *C*-terminal KDEL ER retrieval signal (PDI5) is transferred together with a cysteine protease without the KDEL motif via the Golgi into lytic and protein storage vacuoles. It is suggested that the KDEL-tailed PDI5 functions as a chaperone for transporting proteases from the ER to their site of action and prevents their premature activation. Because PDI5 carries a KDEL ER retrieval signal, it remains to be seen how the ER retrieval signal is silenced during the transport of PDI5 through the Golgi apparatus (Ondzighi et al. 2008). This transport pathway, however, is not prominent for KDEL-CysEPs. Furthermore, there is no need for inhibition by a protein disulfide isomerase during transport or storage, since KDEL CysEPs are present in ricinosomes as the enzymatically inactive pro-form.

Catabolic processes occur in plant senescence/PCD and involve various hydrolytic enzymes for macromolecule degradation. The papain-like cysteine protease RD21 (RESPONSIVE TO DESICCATION 21) contains a *C*-terminal granulin domain (Yamada et al. 2001). RD21 is present in ER bodies and the vacuole (Yamada et al. 2001; Carter et al. 2004). RD21-like proteases play a role in plant immune responses. The *Arabidopsis* nuclease BFN1 is induced during both senescence and developmental PCD (Perez-Amador et al. 2000; Farage-Barhom et al. 2008). BFN1 was localized in filamentous, ER-derived structures as storage compartments scattered throughout the cytoplasm in young leaves (Farage-Barhom et al. 2011). These BFN1-containing filaments clustered and wrapped around the nuclei in the progress of senescence. At the final stage of senescence, when the leaves turned yellow, most of the filaments had disappeared and the nuclease was localized within vesicles that seemed to be derived from the clustered filaments. The filamentous structures may serve as storage and transport compartments bringing the nuclease to its substrate (Farage-Barhom et al. 2011).

Hydrolytic enzymes such as proteases and especially KDEL CysEPs, glucosidases or nucleases are thus stored in ER-derived organelles and are released during developmental PCD or upon biotic stress/wounding. In addition, the concrete function of AtCEP2 stored in ricinosomes or ER bodies might be diverse. The expression of AtCEP2 in root cap formation might suggest a function in the final stage of developmental PCD. The presence of pro-AtCEP2 at the basis of young leaves, in the hypocotyl and in the root elongation zone, on the other hand, might indicate a function for AtCEP2 in loosening the cell wall for extension and/or tissue remodelling by dismantling the extensin scaffold. Due to their ability to cleave at glycosylated hydroxyl-proline, KDEL CysEPs might not only support the cell collapse in the final stage of PCD, but also serve in cell elongation. Ricinosomes and the related KDEL vesicles (KV; Toyooka et al. 2000; Okamoto et al. 2003) seem to be instrumental in the efficient transport of KDEL CysEPs for diverse purposes such as storage mobilization, PCD or tissue remodeling.

Interestingly, AtCEP2 was expressed in the hypocotyl epidermis in cell files containing stomata (and not in the stomata itself) but not in cell files without stomata. In *Arabidopsis*, epidermal cells in the hypocotyl are organized in files that run parallel to the long axis of the seedling. Files consisting of non-protruding cells are placed outside two cortical cell files, whereas that consisting of protruding cells overlay a single cortical cell (Gendreau et al. 1997; Berger et al. 1998). Stomatal development progresses from the upper to the basal part of the hypocotyl with no stomata formed in the basal third of the embryonic stem (Berger et al. 1998). Stomata only develop in epidermal files located outside two cortical cells (Berger et al. 1998; Hung et al. 1998). Only cell elongation, but no cell division occurs in the hypocotyl, except the stomata that derive from mother cells by cell division. Cell wall weakening necessary for cell elongation might be assisted by AtCEP2. It is, however, unclear why ricinosomes were found exclusively in the non-protruding cell files containing the stomata and not in the protruding cell files. The underlying regulation or function needs further investigation.

No obvious *atcep2* knockout phenotype in young seedlings was found, especially not in root cap formation. The *atcep2* knockout phenotype indicates functional redundancy with other proteases necessary for PCD. A dual role in developmental PCD as well as in pathogen defense is established for vacuolar processing enzymes (VPEs) (Hara-Nishimura et al. 2005). VPEs (C13 legumain family) are asparaginyl endopeptidases cleaving their substrate C-terminal to Asp and Asn residues. *Arabidopsis* has four VPE genes (α-VPE, β-VPE, γ-VPE und δ-VPE). VPEs are localized in the vacuole, thus participating in the vacuole-mediated PCD typical for plants. VPE could be a key molecule in plant PCD by disrupting the vacuole. Similar to KDEL-CysEPs, VPEs are specific for plants. Metacaspases are cysteine-dependent proteases found in protozoa, fungi and plants. They lack Asp specificity and cleave their targets after Arg or Lys residues. Metacaspases are essential for normal physiology of non-metazoan organisms; they are involved in programmed cell death, stress and cell proliferation (for review see: Tsiatsiani et al. 2011; Lam and Zhang 2011).

The strong tissue specific expression of *AtCEP1*, *AtCEP2* and *AtCEP3* does not suggest redundancy among *Arabidopsis* KDEL CysEPs (Helm et al. 2008), although this cannot be excluded since *AtCEP1* or *AtCEP3* are slightly up regulated in *atcep2* mutants. Future experiments with double knockout mutant plants expressing the appropriate functional or non-functional reporter-AtCEP fusion proteins should elucidate if the third AtCEP is now expressed in the affected tissues.

## Electronic supplementary material

Below is the link to the electronic supplementary material.

Supplementary material 1 (PDF 1547 kb)

## Abbreviations

3xHA: Three-fold hemagglutinin (HA) tag
CBZ-Phe-Arg-AMC: Fluorescence-quenched peptide
CLSM: Confocal laser scanning microscopy
GFP: Green fluorescent protein
