# Supplementary material for: Ex vivo processing for maturation of Arabidopsis KDEL-tailed cysteine endopeptidase 2 (AtCEP2) pro-enzyme and its storage in endoplasmic reticulum derived organelles
Source: Plant Mol Biol. 2013 Nov 28;84(6):605–20. doi: 10.1007/s11103-013-0157-6 (PMC3950626; doi:10.1007/s11103-013-0157-6)
Supplement: Supplementary file 1 — Supplementary material 1 (PDF 1547 kb) [file 11103_2013_157_MOESM1_ESM.pdf]

Figure S1: Cloning strategy in pGreen 0179 for expression of the fusion protein pre-pro-3xHA-mCherry-AtCEP2-KDEL under control of the endogenous AtCEP2 promoter.

DNA sequence:

GGTACCagtagagtcacttccaagaaactaggaaacatcaaaccacgtcgttgcattttggggatcttatgaaat
acacaagcttatacaaaaaaaaatattataaaaaaatgaacgtatattaattcgtaattacataggcttaaaacc
aagataagatcggtacatgttgaaaatgaaaattattcaatctcctaatagaggtgagacaattatcaattcgaa
actcatacatcaatcataaaagctacaatattttctcaactccatcaattatccgtgggagtcatacaaaaaaaa
aattaaaaaattattacatatatatgtatttttttttcagaaaagtgaaatctctttcgaacatatgaaattat
gaatattcatcaattggcctaggattacgagatcaacgtaaattatttaaataacaaaaataaaaaatatgtttc
cttgcattaggaagcatcattatccaaccacgacgccgggtattaagccaagatccattccaattattcattcat
atctactacctaaaacgacattagctttttataaagaaagtcttaacccacgatcggtgttcatatttaaatatt
acatttgttgttaaacaccaatgttatgcctaacaacttaaataaaaccgccataattgtttttttattgtgtttt
tgaaaaactacttgcccgttgttatcttgtggcctgagtttttttctcttttgtttttaatatatgtagtatga
aaccacgtttcaacaaatgggctgcgccaaatgttcacgcatcttgccttgagaattttgaataacaactaatta
gagattaaggtatggaaatgtacaaaaatcgccacgagatcctttcgtagatccacatggtattaccgggataca
tggagaaacatgttttcttaagatcagtacattactcattaaatggtcgatattagtaaattatttttgtacgta
attgctcggtgcatggtttaagacgtgtgttcttaaaagctactccaattaataatgatgctttcatgtggtgtg
gaattaaaagttttggatgcgtagtggctaatcaaaaaatcataaagttcatagtttaagcttcgaaaatgcaaa
tagcttggggatacaaaagctatctttacaaaaatcttagaaatggtggagtcttacataatcacgtgtgtgatc
caaattatagatgtaatagaagaaatttttcttgtattttcatgtattatatgcatcgcctttatcgtctacaca
tgaccaaaatttttttcccattttcccattttttaatcctatacaaaagaagtttttttttattgtacaaggcattag
gtcaacaatgccatggtgtgaacaactttgtctccacattttggggacatatcttaatctaattatcgctttca
ttacttccctaagcaacaagttgcttcatcttaattttcccattattgtataaaactatccatcaaccctgacaa
atttaaacgatgaaagcctgaattattgataatgatattgatatcattatcatccaaacgatgaaagcctgaatt
attcagttttaaaaggatattcagttttctctcactgtcagctaacttagtcaaatcaaacattcataaatatat
attacacaacatatagatctataagcatgacatttataaaaccatatattataaatcttatgatttattatccta
aactatttagaaagaatatgatgggttccttattcctctcaaatgcgtgtcctagaagttccacaagtctaacca
agacgtatacacaatgatacacaataataattcattttccaaacgttgatggtatttgagtaatttcaaatctct
acagaaaaatatttctctttccctatatatatccgataccatctcattgcactttcaacctcaaacaaataacca
atcaaccaaaaaaaaaaaaaaa**ATG**AAGAAACTTCTTTTGATATTTCTCTTTTCTCTTGTCATTCTCCAAACTGC
TTGTGGATTCGATTACGACGACAAGGAAATAGAAAGCGAAGAGGGCTTATCAACGCTTTATGATAGATGGAGGAG
CCACCACTCCGTTCCTAGGAGTCTAAATGAAAGGGAGAAGAGATTCAACGTTTTCAGACACAATGTCATGCATGT
CCACAATACCAACAAGAAGAACCGATCCTACAAACTTAAGCTCAACAAGTTTGCAGATTTAACAATTAATGAGTT
CAAGAATGCATACACCGGTTCCAACATCAAGCATCACAGAATGTTGCAAGGACCAAAACGCGGCTCAAAACAGTT
CATGTATGATCATGAGAATTTATCCAAATTACCGTCCCTCGAGtacccatacgacgttcctgactatgcgtcact
ctacccctatgacgtaccggattatgcatccctatatccgtatgatgttccagattacgcttctctacgttcctc
tagaggcgtccaccatatgGTCGACatgttgagcaagggcgaggaggataacatggccatcatcaaggagttcat
gcgcttcaaggtgcacatggagggctccgtgaacggccacgagttcgagatcgagggcgagggcgagggccgccc
ctacgagggcacccagaccgccaagctgaaggtgaccaagggtggccccctgcccttcgcctgggacatcctgtc
ccctcagttcatgtacggctccaaggcctacgtgaagcaccccgccgacatccccgactacttgaagctgtcctt
ccccgagggcttcaagtgggagcgcgtgatgaacttcgaggacggcggcgtggtgaccgtgacccaggactcctc
cctgcaggacggcgagttcatctacaaggtgaagctgcgcggcaccaacttcccctccgacggccccgtaatgca
gaagaagaccatgggctgggaggcctcctccgagcggatgtaccccgaggacggcgccctgaagggcgagatcaa
gcagaggctgaagctgaaggacggcggccactacgacgctgaggtcaagaccacctacaaggccaagaagcccgt
gcagctgcccggcgcctacaacgtcaacatcaagttggacatcacctcccacaacgaggactacaccatcgtgga
acagtacgaacgcgccgagggccgccactccaccggcggcatggacgagctgtacaagGGATCCTTACCGTCCTC
CGTTGATTGGAGAAAGAAAGGTGCTGTCACTGAAATCAAGAATCAAGGAAAATGTGGAAGTTGTTGGGCATTCTC
TACCGTAGCAGCTGTTGAGGGAATCAACAAGATCAAGACTAACAAACTGGTCTCACTGTCTGAACAAGAACTAGT
GGATTGTGATACTAAGCAGAATGAAGGTTGTAACGGAGGTCTTATGGAGATTGCATTTGAATTCATCAAGAAGAA
CGGTGGTATCACCACTGAAGATAGCTATCCTTATGAAGGTATCGATGGAAAATGCGACGCTTCAAAGGATAACGG
CGTGCTTGTGACGATAGATGGGCATGAAGATGTACCCGAAAACGACGAAAATGCTCTTCTTAAGGCTGTTGCAAA
CCAACCTGTATCTGTTGCAATTGATGCTGGAAGTTCAGATTTCCAGTTTTATTCAGAGGGAGTGTTTACGGGATC
TTGTGGAACAGAGCTGAACCATGGTGTGGCAGCGGTCGGGTATGGATCTGAGAGGGGGAAAAAGTATTGGATAGT
GAGAAACTCATGGGGAGCTGAATGGGGAGAGGGAGGCTATATAAAGATTGAAAGAGAGATTGATGAACCGGAAGG
GCGTTGCGGTATTGCGATGGAGGCATCGTATCCGATCAAGCTATCGTCGTCTAATCCTACCCCGAAAGACGGTGA
TGTCAAAGATGAGCTC**TAG**atatgtgtacacattgtgtaaaaccatatcaaaggtgatcccaattccttg
ttgattcatgttgttagtttcgtcttcatgatttgattttgttgttggttttgaatttgaataaaaat
gtctcgtgttttctcGCGGCCGC

Primers used for PCR amplification and cloning:

Primer pair 1 for amplification of the promoter and the pre-pro-sequence (2362 bp):

```
Sense:              5’-cattgGGTACCagtagagtcacttccaag-3’
                    Tm ges.: 66°C            Tm: 51°C

Antisense:          5‘-gtaCTCGAGggacggtaatttggataaa-3‘
                    Tm ges.: 65°C            Tm:53°C
```

Primer pair 2 for amplification of the 3xHA tag (132 bp):

```
Sense:              5‘-aaaCTCGAGtacccatacgacgttcctg-3‘
                    Tm ges.: 66°C            Tm:54°C
Antisense:          5’-catGTCGACcatatggtggacgcctct-3’
                    Tm ges.: 65°C            Tm: 52°C
```

Primer pair 3 for amplification of mCerry (714 bp):

```
Sense:              5‘-atgGTCGACatgttgagcaagggcgag-3‘
                    Tm ges.: 65°C            Tm: 52°C
Antisense:          5’-taaGGATCCcttgtacagctcgtccatg-3’
                    Tm ges.: 66°C            Tm: 54°C
```

Primer pair 4 for amplification of AtCEP2 (846 bp):

```
Sense:              5’-aagGGATCCttaccgtcctccgttgat-3’
                    Tm ges.: 65°C            Tm: 52°C
Antisense:          5’-gtgGCGGCCGCgagaaaacacgagacatt-3’
                    Tm ges.: 68°C            Tm: 51°C
```

Protein sequence:

```
MKKLLLIFLFSLVILQTACGFDYDDKEIESEEGLSTLYDRWRSHHSVPRSLNEREKRFNVFRHNVMHVHNTNKKN
RSYKLKLNKFADLTINEFKNAYTGSNIKHHRMLQGPKRGSKQFMYDHENLSKLPSLEYPYDVPDYASLYPYDVPD
YASLYPYDVPDYASLRSSRGVHHMVDMLSKGEEDNMAIIKEFMRFKVHMEGSVNGHEFEIEGEGEGRPYEGTQTA
KLKVTKGGPLPFAWDILSPQFMYGSKAYVKHPADIPDYLKLSFPEGFKWERVMNFEDGGVVTVTQDSSLQDGEFI
YKVKLRGTNFPSDGPVMQKKTMGWEASSERMYPEDGALKGEIKQRLKLKDGGHYDAEVKTTYKAKKPVQLPGAYN
VNIKLDITSHNEDYTIVEQYERAEGRHSTGGMDELYKGSLPSSVDWRKKGAVTEIKNQGKCGSCWAFSTVAAVEG
INKIKTNKLVSLSEQELVDCDTKQNEGCNGGLMEIAFEFIKKNGGITTEDSYPYEGIDGKCDASKDNGVLVTIDG
HEDVPENDENALLKAVANQPVSVAIDAGSSDFQFYSEGVFTGSCGTELNHGVAAVGYGSERGKKYWIVRNSWGAE
WGEGGYIKIEREIDEPEGRCGIAMEASYPIKLSSSNPTPKDGDVKDEL
```

Pre – Pro – 3xHA - mCherry – AtCEP2 - KDEL

Amino acids shaded in white were introduced due to cloning strategy

Figure S2: Cloning strategy in pGreen 0179 for expression of the non-functional fusion protein pre-pro-3xHA-mCherry-KDEL under control of the endogenous AtCEP2 promoter.

DNA sequence:

GGTACCagtagagtcacttccaagaaactaggaaacatcaaaccacgtcgttgcattttggggatcttatgaaat
acacaagcttatacaaaaaaaaatattataaaaaaatgaacgtatattaattcgtaattacataggcttaaaacc
aagataagatcggtacatgttgaaaatgaaaattattcaatctcctaatagaggtgagacaattatcaattcgaa
actcatacatcaatcataaaagctacaatattttctcaactccatcaattatccgtgggagtcatacaaaaaaaa
aattaaaaaaattattacatatatatgtattttttttcagaaaagtgaaatctctttcgaacatatgaaattat
gaatattcatcaattggcctaggattacgagatcaacgtaaattatttaaataacaaaaataaaaaatatgtttc
cttgcattaggaagcatcattatccaaccacgacgccgggtattaagccaagatccattccaattattcattcat
atctactacctaaaacgacattagctttttataaagaaagtcttaacccacgatcggtgttcatatttaaatatt
acatttgttgttaaacaccaatgttatgcctaacaacttaaataaaaccgccataattgtttttttattgtgtttt
tgaaaaactacttgcccgttgttatcttgtggcctgagttttttttctcttttgtttttaatatatgtagtatga
aaccacgtttcaacaaatgggctgcgccaaatgttcacgcatcttgccttgagaattttgaataacaactaatta
gagattaaggtatggaaatgtacaaaaatcgccacgagatcctttcgtagatccacatggtattaccgggataca
tggagaaacatgttttcttaagatcagtacattactcattaaatggtcgatattagtaaattatttttgtacgta
attgctcggtgcatggtttaagacgtgtgttcttaaaagctactccaattaataatgatgctttcatgtggtgtg
gaattaaaagttttggatgcgtagtggctaatcaaaaaatcataaagttcatagtttaagcttcgaaaatgcaaa
tagcttggggatacaaaagctatctttacaaaaatcttagaaatggtggagtcttacataatcacgtgtgtgatc
caaattatagatgtaatagaagaaatttttcttgtattttcatgtattatatgcatcgcctttatcgtctacaca
tgaccaaaatttttttcccattttcccatttttaatcctatacaaaagaagttttttttattgtacaaggcattag
gtcaacaatgccatggtgtgaacaactttgtctccacattttggggacatatcttaatctaattatcgctttca
ttacttccctaagcaacaagttgcttcatcttaattttcccattattgtataaaactatccatcaaccctgacaa
atttaaacgatgaaagcctgaattattgataatgatattgatatcattatcatccaaacgatgaaagcctgaatt
attcagttttaaaaggatattcagttttctctcactgtcagctaacttagtcaaatcaaacattcataaatatat
attacacaacatatagatctataagcatgacatttataaaaccatatattataaatcttatgatttattatccta
aactatttagaaagaatatgatgggttccttattcctctcaaatgcgtgtcctagaagttccacaagtctaacca
agacgtatacacaatgatacacaataataattcattttccaaacgttgatggtatttgagtaatttcaaatctct
acagaaaaatatttctctttccctatatatatccgataccatctcattgcactttcaacctcaaacaaataacca
atcaaccaaaaaaaaaaaaaaaa**ATG**AAGAAACTTCTTTTGATATTTCTCTTTTCTCTTGTCATTCTCCAAACTGC
TTGTGGATTCGATTACGACGACAAGGAAATAGAAAGCGAAGAGGGCTTATCAACGCTTTATGATAGATGGAGGAG
CCACCACTCCGTTCCTAGGAGTCTAAATGAAAGGGAGAAGAGATTCAACGTTTTCAGACACAATGTCATGCATGT
CCACAATACCAACAAGAAGAACCGATCCTACAAACTTAAGCTCAACAAGTTTGCAGATTTAACAATTAATGAGTT
CAAGAATGCATACACCGGTTCCAACATCAAGCATCACAGAATGTTGCAAGGACCAAAACGCGGCTCAAAACAGTT
CATGTATGATCATGAGAATTTATCCAAATTACCGTCCCTCGAGtacccatacgacgttcctgactatgcgtcact
ctacccctatgacgtaccggattatgcatccctatatccgtatgatgttccagattacgcttctctacgttcctc
tagaggcgtccaccatatgGTCGACatgttgagcaagggcgaggaggataacatggccatcatcaaggagttcat
gcgcttcaaggtgcacatggagggctccgtgaacggccacgagttcgagatcgagggcgagggcgagggccgccc
ctacgagggcacccagaccgccaagctgaaggtgaccaagggtggccccctgcccttcgcctgggacatcctgtc
ccctcagttcatgtacggctccaaggcctacgtgaagcaccccgccgacatccccgactacttgaagctgtcctt
ccccgagggcttcaagtgggagcgcgtgatgaacttcgaggacggcggcgtggtgaccgtgacccaggactcctc
cctgcaggacggcgagttcatctacaaggtgaagctgcgcggcaccaacttcccctccgacggccccgtaatgca
gaagaagaccatgggctgggaggcctcctccgagcggatgtaccccgaggacggcgccctgaagggcgagatcaa
gcagaggctgaagctgaaggacggcggccactacgacgctgaggtcaagaccacctacaaggccaagaagcccgt
gcagctgcccggcgcctacaacgtcaacatcaagttggacatcacctcccacaacgaggactacaccatcgtgga
acagtacgaacgcgccgagggccgccactccaccggcggcatggacgagctgtacaagGGATCCGGTGATGTCAA
AGATGAGCTCTAGatatgtgtacacattgtgtaaaaccatatcaaaggtgatcccaattccttgttgat
tcatgttgttagtttcgtcttcatgatttgattttgttgttggttttgaatttgaataaaaatgtctc
gtgttttctcGCGGCCGC

Primers used for PCR amplification and cloning :

Primer pair 1 for amplification of the promoter and the pre-pro-sequence (2362 bp):

Sense:	cattgGGTACCagtagagtcacttccaag	
	Tm ges.: 66°C	Tm: 51°C
Antisense:	gtaCTCGAGggacggtaatttggataaa	
	Tm ges.: 65°C	Tm:53°C

Primer pair 2 for amplification oft the 3xHA tag (132 bp):

Sense:	aaaCTCGAGtacccatacgacgttcctg	
	Tm ges.: 66°C	Tm:54°C
Antisense:	catGTCGACcatatggtggacgcctct	
	Tm ges.: 65°C	Tm: 52°C

Primer pair 3 for amplification of mCerry (714 bp):

Sense:	atgGTCGACatgttgagcaagggcgag	
	Tm ges.: 65°C	Tm: 52°C
Antisense:	taaGGATCCcttgtacagctcgtccatg	
	Tm ges.: 66°C	Tm: 54°C

Primer pair 4 for amplification oft he KDEL-3´UTR (178 bp):

Sense-kdel	aagGGATCCGGTGATGTCAAAGATGAGC	
	Tm ges.: 65°C	Tm: 54°C
Antisense:	gtgGCGGCCGCgagaaaacacgagacatt	
	Tm ges.: 68°C	Tm: 51°C

Protein sequence:

MKKLLLIFLFSLVILQTACGFDYDDKEIESEEGLSTLYDRWRSHHSVPRSLNEREKRFNVFRHNVMHVHNTNKKN
RSYKLKLNKFADLTINEFKNAYTGSNIKHHRMLQGPKRGSKQFMYDHENLSKLPSLEYPYDVPDYASLYPYDVPD
YASLYPYDVPDYASLRSSRGVHHMVDMLSKGEEDNMAIIKEFMRFKVHMEGSVNGHEFEIEGEGEGRPYEGTQTA
KLKVTKGGPLPFAWDILSPQFMYGSKAYVKHPADIPDYLKLSFPEGFKWERVMNFEDGGVVTVTQDSSLQDGEFI
YKVKLRGTNFPSDGPVMQKKTMGWEASSERMYPEDGALKGEIKQRLKLKDGGHYDAEVKTTYKAKKPVQLPGAYN
VNIKLDITSHNEDYTIVEQYERAEGRHSTGGMDELYKGSGDVKDEL

Pre – Pro – 3xHA – mCherry – KDEL

Amino acids shaded in white were introduced due to cloning strategy

Figure S3: Anti-peptide antibodies are specific for the respective AtCEP pro-enzymes. A: Alignment of KDEL-CysEPs from *Ricinus communis* and *Arabidopsis thaliana* (adapted from Helm et al. 2008). The arrow ↓ indicates the cleavage site of the pre-sequence that is co-translationally removed during transfer into the ER; the double arrow ↓↓ indicates cleavage of the pro-sequence and the last 10 amino acids including the KDEL motif during maturation to the active enzyme (Schmid et al. 1998; Than et al. 2004). Conserved amino acids are shaded. Synthetic peptides used for immunization of rabbits are indicated in red. **B: The anti-peptide antibodies are specific for pro-AtCEP1, pro-AtCEP2 and pro-AtCEP3, respectively, overexpressed in *E.coli*.** Lane 1 and 2, protein extracts from *E.coli* harbouring the empty vector un-induced or induced, respectively. Lane 3-5, protein extracts from *E. coli* over-expressing AtCEP1 (lane 3) AtCEP2 (lane 4) and AtCEP3 (lane5). Lane 5, castor bean CysEP from isolated ricinosomes. M, molecular weight marker. The antibodies used for decoration are indicated above the westernblot.

A

```
                   1                                ↓                                                                                   100
Ricinus communis~~MQKFILLA LSLALVLA.. .ITESFDFHE KELESEESLW GLYERWRSHH TVSR...... ...SLHEKQK RFNVFKHNAM HVHNAN.KMD KPYKLKLNKF
Arabidopsis       ~~MKRFIVLA LCMLMVLE.. .TTKGLDFHN KDVESENSLW ELYERWRSHH TVAR...... ...SLEEKAK RFNVFKHNVK HIHETN.KKD KSYKLKLNKF
Arabidopsis       ~~MKKLLLIF LFSLVILQ.. .TACGFDYDD KEIESEEGLS TLYDRWRSHH SVPR...... ...SLNEREK RFNVFRHNVM HVHNTN.KKN RSYKLKLNKF
Arabidopsis       ~~MKLFFIVL ISFLSLLQ.. .ASKGFDFDE KELETEENVW KLYERWRGHH SVSR...... ...ASHEAIK RFNVFRHNVL HVHRTN.KKN KPYKLKINRF

                   101                                                 ↓↓                                                        200
R. communis        ADMTNHEFRN TYSGSKVKHH RMFRGGPRGN GT...FMY.. EKVDTVPA.S VDWRKKGAVT SVKDQGQC.G SCWAFSTIVA VEGINQIKTN KLVSLSEQEL
Arabidopsis        GDMTSEEFRR TYAGSNIKHH RMFQGEKKAT KS...FMY.. ANVNTLPT.S VDWRKNGAVT PVKNQGQC.G SCWAFSTVVA VEGINQIRTK KLTSLSEQEL
Arabidopsis        ADLTINEFKN AYTGSNIKHH RMLQGPKRGS KQ...FMYDH ENLSKLPS.S VDWRKKGAVT EIKNQGKCGG SCWAFSTVAA VEGINKIKTN KLVSLSEQEL
Arabidopsis        ADITHHEFRS SYAGSNVKHH RMLRGPKRGS GG...FMY.. ENVTRVPS.S VDWREKGAVT EVKNQQDC.G SCWAFSTVAA VEGINKIRTN KLVSLSEQEL

                   201                                                                                                           300
R. communis        VDCDTDQNQG CNGGLMDYAF EFIKQRGGIT TEANYPYEAY D.GTCDVSKE NAPAVSIDGH ENVPENDENA LLKAVANQPV SVAIDAGGSD FQFYSEGVFT
Arabidopsis        VDCDTNQNQG CNGGLMDLAF EFIKEKGGLT SELVYPYKAS D.ETCDTNKE NAPVVSIDGH EDVPKNSEDD LMKAVANQPV SVAIDAGGSD FQFYSEGVFT
Arabidopsis        VDCDTKQNEG CNGGLMEIAF EFIKKNGGIT TEDSYPYEGI D.GKCDASKD NGVLVTIDGH EDVPENDENA LLKAVANQPV SVAIDAGSSD FQFYSEGVFT
Arabidopsis        VDCDTEENQG CAGGLMEPAF EFIKNNGGIK TEETYPYDSS DVQFCRANSI GGETVTIDGH EHVPENDEEE LLKAVAHQPV SVAIDAGSSD FQLYSEGVFI

                   301                                                                                  ↓↓        387
R. communis        GSCGTELDHG VAIVGYGTTI DGTKYWTVKN SWGPEWGEKG YIRMERGI.S DKEGLCGIAM EASYPIKKSS NNPSGI....K SSPKDEL*CysEPAF050756
Arabidopsis        GRCGTELNHG VAVVGYGTTI DGTKYWIVKN SWGEEWGEKG YIRMQRGI.R HKEGLCGIAM EASYPLKNSN TNPSRLS...L DSLKDEL*CEP1 AB024031
Arabidopsis        GSCGTELNHG VAAVGYG.SE RGKKYWIVRN SWGAEWGEGG YIKIEREI.D EPEGRCGIAM EASYPIKLSS SNPTPKD.... GDVKDEL*CEP2 AL049659
Arabidopsis        GECGTQLNHG VVIVGYGETK NGTKYWIVRN SWGPEWGEGG YVRIERGI.S ENEGRCGIAM EASYPTKLSS .TPSTHESVVR DDVKDEL*CEP3 AL049659
```

B

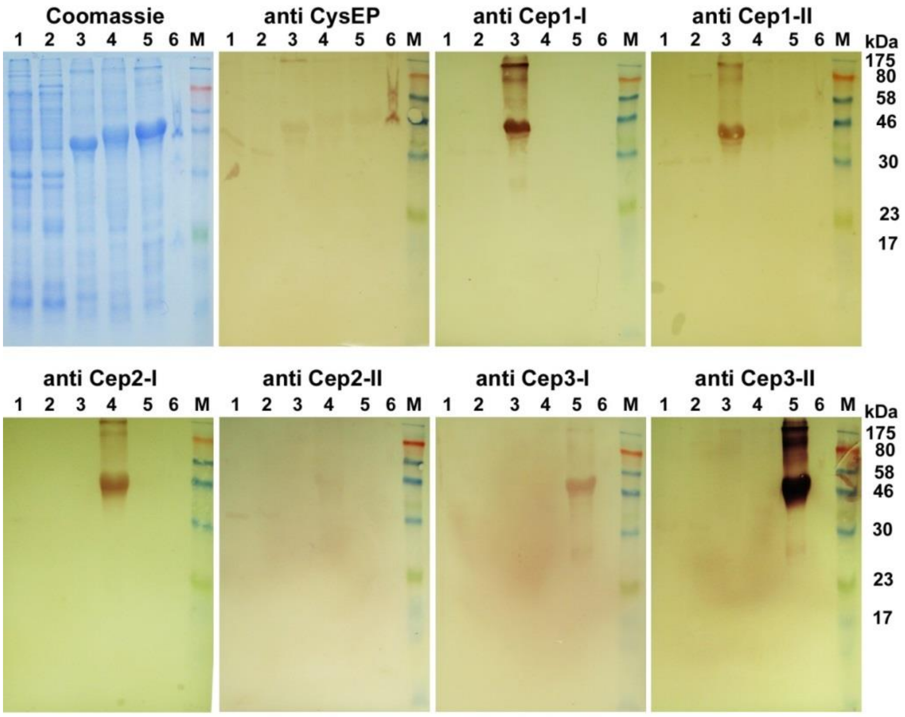

Figure S4: Antibodies directed against the mature subunit of AtCEP1 cross-react with AtCEP2

A: Cloning strategy. M, L and E (shaded in green) and the His-tag (yellow) were added by cloning.

AtCEP1 (636bp)

```
GGAGCCGTCACTCCTGTCAAAAACCAAGGCCAATGCGGGAGTTGTTGGGCGTTTTCAACAGTTGTTGCGGTGGAA
GGGATCAACCAAATAAGAACTAAAAAGCTGACATCACTCTCAGAGCAAGAGCTAGTAGATTGTGATACAAACCAG
AACCAAGGATGCAATGGAGGTCTAATGGACCTTGCTTTTGAGTTCATCAAGGAGAAAGGAGGACTCACAAGTGAG
CTAGTGTACCCTTACAAGGCTTCTGATGAAACTTGTGACACAAACAAAGAAAATGCTCCGGTAGTTTCAATCGAT
GGACACGAAGATGTTCCTAAGAACAGCGAGGATGATCTAATGAAAGCTGTTGCTAATCAGCCTGTTTCTGTTGCT
ATTGATGCTGGAGGCTCAGACTTTCAATTCTACTCCGAGGGAGTGTTTACCGGGAGATGCGGAACAGAGCTAAAC
CATGGAGTTGCGGTAGTAGGGTATGGAACAACGATAGACGGAACAAAGTATTGGATTGTTAAGAATTCATGGGGA
GAGGAATGGGGAGAGAAAGGATACATAAGAATGCAGAGAGGGATTCGTCATAAAGAAGGACTTTGTGGTATTGCA
ATGGAAGCTTCTTATCCTCTCAAGAACTCCAACACT
```

```
CEP1 sense        5'-agaCATATGGGAGCCGTCACTCCTGTCAA-3`
CEP1 anti-sense   5'-agaCTCGAGAGTGTTGGAGTTCTTGAGAGG-3'
```

Protein:

MGAVTPVKNQGQCGSCWAFSTVVAVEGINQIRTKKLTSLSEQELVDCDTNQNQGCNGGLMDLAFEFIKEKGGLTSELVYPYKA
SDETCDTNKENAPVVSIDGHEDVPKNSEDDLMKAVANQPVSVAIDAGGSDFQFYSEGVFTGRCGTELNHGVAVVGYGTTIDG
TKYWIVKNSWGEEWGEKGYIRMQRGIRHKEGLCGIAMEASYPLKNSNTLEHHHHHH*

AtCEP2 (630bp)

```
GGTGCTGTCACTGAAATCAAGAATCAAGGAAAATGTGGAAGTTGTTGGGCATTCTCTACCGTAGCAGCTGTTGAG
GGAATCAACAAGATCAAGACTAACAAACTGGTCTCACTGTCTGAACAAGAACTAGTGGATTGTGATACTAAGCAG
AATGAAGGTTGTAACGGAGGTCTTATGGAGATTGCATTTGAATTCATCAAGAAGAACGGTGGTATCACCACTGAA
GATAGCTATCCTTATGAAGGTATCGATGGAAAATGCGACGCTTCAAAGGATAACGGCGTGCTTGTGACGATAGAT
GGGCATGAAGATGTACCCGAAAACGACGAAAATGCTCTTCTTAAGGCTGTTGCAAACCAACCTGTATCTGTTGCA
ATTGATGCTGGAAGTTCAGATTTCCAGTTTTATTCAGAGGGAGTGTTTACGGGATCTTGTGGAACAGAGCTGAAC
CATGGTGTGGCAGCGGTCGGGTATGGATCTGAGAGGGGGAAAAAGTATTGGATAGTGAGAAACTCATGGGGAGCT
GAATGGGGAGAGGGAGGCTATATAAAGATTGAAAGAGAGATTGATGAACCGGAAGGGCGTTGCGGTATTGCGATG
GAGGCATCGTATCCGATCAAGCTATCGTCG
```

```
CEP2 sense        5'-agaCATATGGGTGCTGTCACTGAAATCAAGA-3'
CEP2 anti-sense   5'-agaCTCGAGCGACGATAGCTTGATCGGAT-3'
```

Protein:

MGAVTEIKNQGKCGSCWAFSTVAAVEGINKIKTNKLVSLSEQELVDCDTKQNEGCNGGLMEIAFEFIKKNGGITT
EDSYPYEGIDGKCDASKDNGVLVTIDGHEDVPENDENALLKAVANQPVSVAIDAGSSDFQFYSEGVFTGSCGTEL
NHGVAAVGYGSERGKKYWIVRNSWGAEWGEGGYIKIEREIDEPEGRCGIAMEASYPIKLSSLEHHHHHH*

B: Westernblot analysis

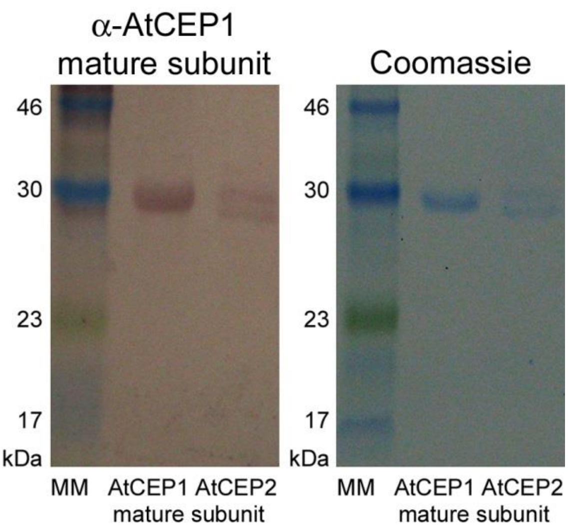

Figure S5: AtCEP2 accepts proline near the cleavage site thus exhibiting the broad substrate specificity typical for KDEL-CysEPs.

Amino acids of the entire beta-casein sequence (224 aa) including the pre-sequence (amino acid residues 1-15) and the mature beta-casein (amino acid residues 16-224) with the cleavage sites indicated by arrows; proline residues are shown in red.

MKVLILACLVALALA-
REL↓E↓ELN↓VPGEIVESLSSSEESITRINKK↓IE↓KFQ↓S↓EEQQQTEDELQ↓DKIHPFA↓Q↓TQS↓LV↓YP↓F
PGPIP↓N↓S↓L↓PQ↓NIPPLT↓Q↓TPVV↓V↓PPFL↓QPEVM↓G↓VS↓KVK↓E↓A↓MAPKH↓K↓E↓MPFPK↓YPVE
PFT↓ES↓QS↓LT↓LT↓DVENLH↓LPLPLL↓Q↓SWM↓H↓QPH↓QPLPPTVM↓FPPQSVLS↓LS↓Q↓SKV↓LPVPQ↓
K↓A↓VPYPQ↓R↓D↓MPIQ↓AF↓L↓L↓Y↓Q↓EPVL↓GPVR↓GPFPIIV

Cleavage sites in beta-casein ↓ identified by amino acid sequencing alone or in combination with MALDI-TOF and summary of amino acids found in positions P2, P2, P1' and P2'

MKVLILACLVALALA	KHKE↓MPFP
-REL↓EELN	PFPK↓YPVE
RELE↓ELNVP	EPFT↓ESQS
EELN↓VPGEIVESLSSSEESITR	FTES↓QSLT
INKK↓IEKF	ESQS↓LTLT
KKIE↓KFQS	QSLT↓LTDV
EKFQ↓SEEQ	LTLT↓DVEN
KFQS↓EEQQQTE	ENLH↓LPLP
DELQ↓DKIH	LPLL↓QSWM
HPFA↓QTQS	PLLQ↓SWMH
PFAQ↓TQSL	QSWM↓HQPH
QTQS↓LVYP	SWMH↓QPHQ
QSLV↓YPFP	HQPH↓QPLP
LVYP↓FPGP	PTVM↓FPPQ
GPIP↓NSLPQ	SVLS↓LSQS
PIPN↓SLPQ	LSLS↓QSKV
IPNS↓LPQN	SLSQ↓SKVL
PNSL↓PQNI	QSKV↓LPVP
SLPQ↓NIPP	PVPQ↓KAVP
PPLT↓QTPV	VPQK↓AVPY
PLTQ↓TPVV	PQKA↓VPYP
TPVV↓VPPF	PYPQ↓RDMP
PVVV↓PPFL	YPQR↓DMPI
PPFL↓QPEV	PQRD↓MPIQ
PEVM↓GVSK	MPIQ↓AFLL
EVMG↓VSKV	IQAF↓LLYQ
MGVS↓KVKE	QAFL↓LYQE
SKVK↓EAMA	AFLL↓YQEP
KVKE↓AMAP	FLLY↓QEPV
VKEA↓MAPK	LLYQ↓EPVL
APKH↓KEMP	EPVL↓GPVR
PKHK↓EMPF	GPVR↓GPFPIIV

P2	P1	P1'	P2'
L (14x)	Q (11x)	Q (9x)	P (20x)
V (8x)	S (8x)	L (9x)	S (7x)
K (6x)	L (7x)	E (7x)	E (6x)
P (6x)	K (5x)	K (4x)	V (5x)
Q (5x)	H (4x)	V (4x)	Q (4x)
F (5x)	E (4x)	S (4x)	T (4x)
I (3x)	T (4x)	M (3x)	L (4x)
E (3x)	V (4x)	A (3x)	M (3x)
M (2x)	A (3x)	G (3x)	A (3x)
Y (2x)	M (3x)	Y (3x)	F (2x)
S (2x)	P (2x)	D (3x)	Y
A (2x)	N (2x)	P (2x)	K
W	R (2x)	F (2x)	D
H	D	T (2x)	I
R	F	N (2x)	W
T	Y	I	
N	G	R	
		H	

Residues in black correspond to the primary sequence, and those shown in red are replicates to illustrate the cleavage site from P4 to P4'.

distibution	P2	P1	P1'	P2'
nonpolar	65 %	32 %	38 %	62 %
basic	13 %	17 %	10 %	2 %
acidic	5 %	8 %	16 %	11 %
polar, uncharched	17 %	43 %	36 %	25 %

Figure S6: Expression of *AtCEP1* (At5g50260), *AtCEP2* (At3g48340) and *AtCEP3* (At3g48350) as visualized by the "root expression map visualizer [www.arexdb.org/]" shown in red (middle and right) and the *Arabidopsis* root template (left). The www.arexdb.org database attributes the observed expression for the KDEL peptidase(s) to *AtCEP1* (middle) and *AtCEP3* (right). No data set is available for AtCEP2 in the ATH1 database. Comparison with our results from P_{CEP1}::*GUS*, P_{CEP2}::*GUS* and P_{CEP3}::*GUS* plants (Helm et al., 2008) and P_{CEP2}::pre-pro-3xHA-mCherry-AtCEP2-KDEL, however, suggests that the *AtCEP2* expression pattern has erroneously been attributed to *AtCEP1* (At5g50260, probe ID 248545_at) or AtCEP3 (At3g48350; probe ID_252368_at).

"AtCEP1" "AtCEP3"

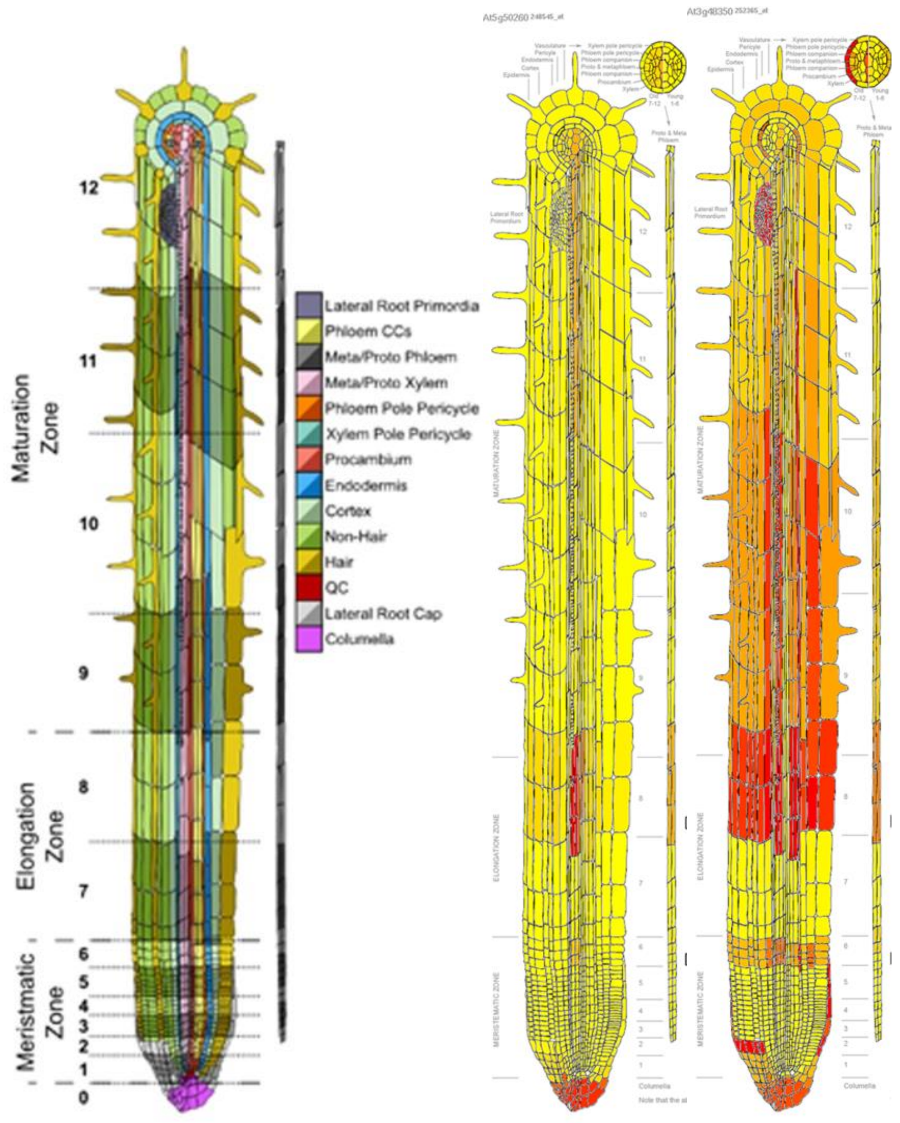

Figure S7: *AtCEP1* and *AtCEP3* transcripts are slightly up-regulated in *cep2*. Relative gene expression of *AtCEP1*, *AtCEP2* and *AtCEP3* in wild-type (WT) and *cep2* seedlings. Total RNA was extracted from 7-day-old seedlings. Expression levels were normalized to the reference gene *ACT8* and the expression in wild type was set to 1 in each experiment. Error bars: SEM.

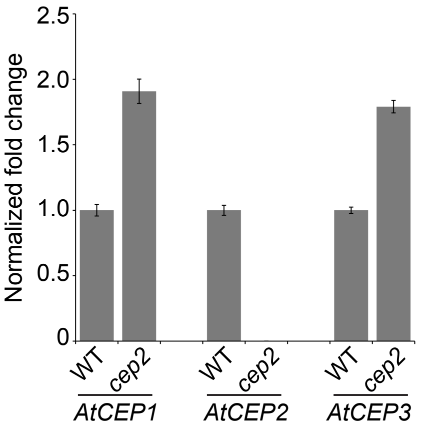

Table S1: Beta-casein peptides obtained by cleavage with AtCEP2

aa-position in beta-casein	Peptide sequence
16 - 22 N-term	-.RELEELN‖V
50 - 68	Q‖SEEQQQTEDELQDKIHPFA‖Q
51 - 68	S‖EEQQQTEDELQDKIHPFA‖Q
62 - 68	Q‖DKIHPFA‖Q
62 - 69	Q‖DKIHPFAQ‖T
73 - 82	S‖LVYPFPGPIP‖N
73 - 87	S‖LVYPFPGPIHNSLPQ‖N
73 - 93	S‖LVYPFPGPIHNSLPQNIPPLT‖Q
75 - 82	V‖YPFPGPIP‖N
75 - 83	V‖YPFPGPIPN‖S
75 - 84	V‖YPFPGPIPNS‖L
75 - 87	V‖YPFPGPIPNSLPQ‖N
75 - 93	V‖YPFPGPIPNSLPQNIPPLT‖Q
77 - 87	P‖FPGPIPNSLPQ‖N
94 - 108	T‖QTPVVVPPFLQPEVM‖G
94 - 103	T‖QTPVVVPPFL‖Q
94 - 111	T‖QTPVVVPPFLQPEVMGVS‖K
95 - 103	Q‖TPVVVPPFL‖Q
100 - 108	V‖PPFLQPEVM‖G
100 - 109	V‖PPFLQPEVMG‖V
100 - 111	V‖PPFLQPEVMGVS‖K
110 - 135	G‖VSKVKEAMAPKHKEMPFPKYPVEPFT‖E
110 - 141	G‖VSKVKEAMAPKHKEMPFPKYPVEPFTESQSLT‖L
112 - 128	S‖KVKEAMAPKHKEMPFPK‖Y
112 - 135	S‖KVKEAMAPKHKEMPFPKYPVEPFT‖E
112 - 137	S‖KVKEAMAPKHKEMPFPKYPVEPFTES‖Q
112 - 141	S‖ .KVKEAMAPKHKEMPFPKYPVEPFTESQSLT‖L
115 - 135	K‖EAMAPKHKEMPFPKYPVEPFT‖E
115 - 141	K‖EAMAPKHKEMPFPKYPVEPFTESQSLT‖L
116 - 135	E‖AMAPKHKEMPFPKYPVEPFT‖E
116 - 141	E‖AMAPKHKEMPFPKYPVEPFTESQSLT‖L
117 - 135	A‖MAPKHKEMPFPKYPVEPFT‖E
122 - 128	H‖KEMPFPK‖Y
122 - 135	H‖KEMPFPKYPVEPFT‖E
122 - 141	H‖KEMPFPKYPVEPFTESQSLT‖L
123 - 135	K‖EMPFPKYPVEPFT‖E
123 - 137	K‖EMPFPKYPVEPFTES‖Q
123 - 141	K‖EMPFPKYPVEPFTESQSLT‖L
124 - 135	E‖MPFPKYPVEPFT‖E
129 - 135	K‖YPVEPFT‖E
140 - 156	S‖LTLTDVENLHLPLPLLQ‖S
144 - 149	T‖DVENLH‖L
144 - 155	T‖DVENLHLPLPLL‖Q
144 - 166	T‖DVENLHLPLPLLQ‖S
150 - 156	H‖LPLPLLQ‖S
160 - 171	M‖HQPHQPLPPTVM‖F
160 - 179	M‖HQPHQPLPPTVMFPPQSVLS‖L
161 - 171	H‖QPHQPLPPTVM‖F
161 - 179	H‖QPHQPLPPTVMFPPQSVLS‖L
164 - 171	H‖QPLPPTVM‖F
182 - 191	S‖QSKVLPVPQK‖A
182 - 192	S‖QSKVLPVPQKA‖V
182 - 198	S‖QSKVLPVPQKAVPYPQR‖D
182 - 203	S‖QSKVLPVPQKAVPYPQRDMPIQ‖A
182 - 205	S‖QSKVLPVPQKAVPYPQRDMPIQAF‖L
182 - 206	S‖QSKVLPVPQKAVPYPQRDMPIQAFL‖L
182 - 207	S‖QSKVLPVPQKAVPYPQRDMPIQAFLL‖Y

183 - 191		Q‖SKVLPVPQK‖A
186 - 203		V‖LPVPQKAVPYPQRDMPIQ‖A
191 - 198		Q‖KAVPYPQR‖D
191 - 203		Q‖KAVPYPQRDMPIQ‖A
192 - 198		K‖AVPYPQR‖D
192 - 199		K‖AVPYPQRD‖M
192 - 203		K‖AVPYPQRDMPIQ‖A
192 - 205		K‖AVPYPQRDMPIQAF‖L
192 - 206		K‖AVPYPQRDMPIQAFL‖L
192 - 224	C-term	K‖AVPYPQRDMPIQAFLLYQEPVLGPVRGPFPIIV.-
193 - 198		A‖VPYPQR‖D
193 - 199		A‖VPYPQRD‖M
193 - 203		A‖VPYPQRDMPIQ‖A
198 - 203		Q‖RDMPIQ‖A
200 - 224	C-term	D‖MPIQAFLLYQEPVLGPVRGPFPIIV.-
204 - 224	C-term	Q‖AFLLYQEPVLGPVRGPFPIIV.-
207 - 224	C-term	L‖LYQEPVLGPVRGPFPIIV.-
208 - 217		L‖YQEPVLGPVR‖G
208 - 224	C-term	L‖YQEPVLGPVRGPFPIIV.-
209 - 217		Y‖QEPVLGPVR‖G
210 - 217		Q‖EPVLGPVR‖G
214 - 224	C-term	L‖GPVRGPFPIIV.-
218 - 224	C-term	R‖GPFPIIV.-
